# Dynamics of networks during absence seizure's on- and offset in rodents and man

**DOI:** 10.3389/fphys.2015.00016

**Published:** 2015-02-05

**Authors:** Annika Lüttjohann, Gilles van Luijtelaar

**Affiliations:** ^1^Donders Centre for Cognition, Donders Instiute for Brain, Cognition and Behaviour, Radboud University NijmegenNijmegen, Netherlands; ^2^Institute of Physiology I, Westfälische Wilhelms-University MünsterMünster, Germany

**Keywords:** cortico-thalamo-cortical system, seizure dynamics, network interactions, Granger causality, pairwise-phase-consistency, non-linear-association analysis, genetic absence models, childhood absence epilepsy

## Abstract

Network mechanisms relevant for the generation, maintenance and termination of spike-wave discharges (SWD), the neurophysiological hallmark of absence epilepsy, are still enigmatic and widely discussed. Within the last years, however, improvements in signal analytical techniques, applied to both animal and human fMRI, EEG, MEG, and ECoG data, greatly increased our understanding and challenged several, dogmatic concepts of SWD. This review will summarize these recent data, demonstrating that SWD are not primary generalized, are not sudden and unpredictable events. It will disentangle different functional contributions of structures within the cortico-thalamo-cortical system, relevant for the generation, generalization, maintenance, and termination of SWD and will present a new “network based” scenario for these oscillations. Similarities and differences between rodent and human data are presented demonstrating that in both species a local cortical onset zone of SWD exists, although with different locations; that in both some forms of cortical and thalamic precursor activity can be found, and that SWD occur through repetitive cyclic activity between cortex and thalamus. The focal onset zone in human data could differ between patients with varying spatial and temporal dynamics; in rats the latter is still poorly investigated.

## Introduction

Absence epilepsy is a neurological disorder mostly found in children between 4 and 12 years of age. It is characterized by sudden and frequent, unpredictable lapses of consciousness, which are accompanied by rhythmic, highly stereotyped, pathological (2.5–4 Hz) oscillations, called spike and wave discharges (SWD), which can be recorded in the electroencephalogram of patients. Given their fast bilateral synchronization, SWD have long been regarded as a primary generalized type of seizure, which seemingly started at all locations at the same time. As a consequence, generation mechanisms and onset location have heavily been debated during the last decades.

There is wide agreement that SWD are generated within the cortico-thalamo-cortical system and that the integrity of this network is a prerequisite for the occurrence of the “full blown,” bilateral, symmetrical SWD (Jasper and Fortuyn, 1947; Gloor et al., 1990; Danober et al., 1998; Seidenbecher et al., 1998; McCormick and Contreras, 2001; Coenen and van Luijtelaar, 2003; Meeren et al., 2005; Pinault and O'Brien, 2005; van Luijtelaar and Sitnikova, 2006; Huguenard and McCormick, 2007). However, the relative contribution and interactions between the various elements of the system, to SWD generation and maintenance, remain enigmatic. Therefore, it comes to no surprise that among the several theories on SWD origin (Meeren et al., 2005) the most popular two (the cortico reticular theory, Gloor, 1968 and cortical focus theory Meeren et al., 2002; Polack et al., 2007), are “network theories.” That is, both theories attribute a functional role for SWD occurrence to the interaction between the cortex and thalamus. However, the functional weighing that these theories give to cortex and thalamus respectively, differs extensively.

The cortico-reticular theory (Gloor, 1968) assumes that a generally hyperexcitable cortex transforms normal sleep spindles, generated in the intrathalamic circuitry (consisting of the Reticular thalamic nucleus (RTN) and Thalamo-Cortical (TC) relay cells), into pathological SWD. By this, the cortico-reticular theory assigns the thalamus with the function of the rhythm generator, whereas the cortex is seen as a general hyperexcitable transformer from normal into pathological oscillations.

**Figure 1:**
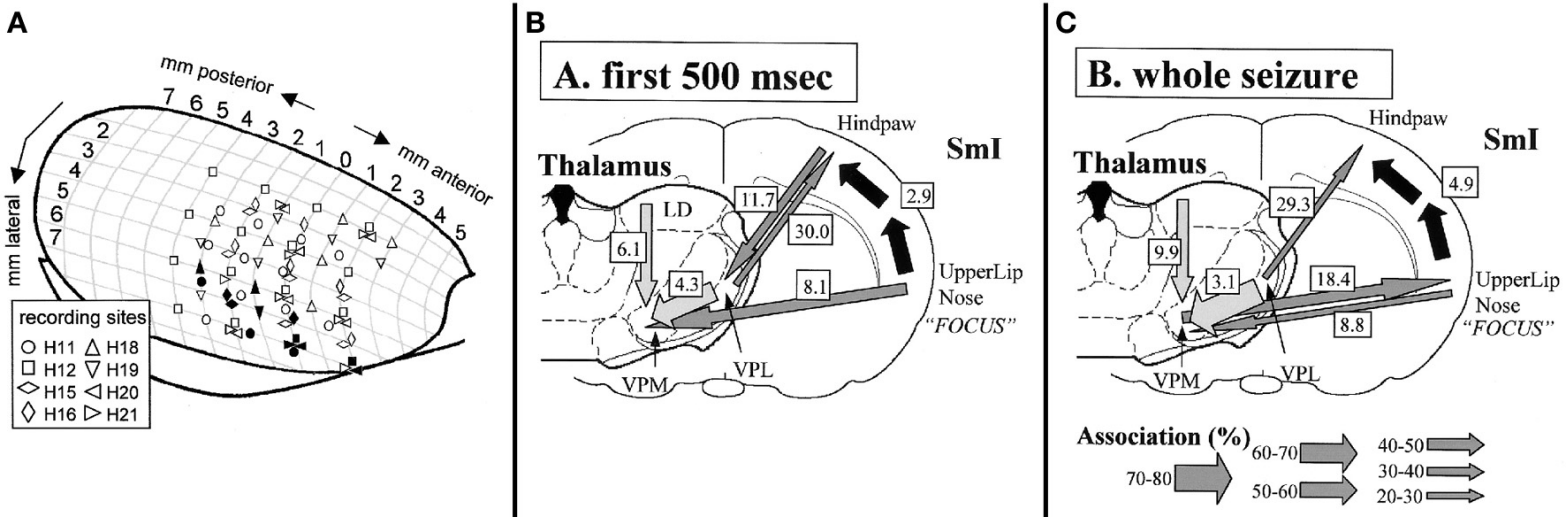
**Electrode locations (A) and summary of results (B+C) obtained in the study by Meeren et al. (2002) inspiring the formulation of the cortical focus theory**. In A different symbol-shapes indicate the recording positions of 8 different rats (H11 to H21, respectively) across anterior-posterior and medial lateral coordinates of one hemisphere of a rat brain. Filled symbols represent the SWD onset sites of the different rats as determined by non-linear association analysis. It can be noted that that the onset zone of the individual rats are exclusively positioned in the most ventrolateral recording positions. In **(B,C)** strength and direction of coupling are represented by arrows, with the thickness of the arrow representing a different degree of coupling strength. It can be noted that during the first 500 ms of the SWD, the S1-upper lip region drives all other recorded areas **(B)**, while thereafter **(C)** a bidirectional crosstalk between cortical focus and thalamus can be detected. Figure adapted from Meeren et al. (2002).

The cortical focus theory was inspired by findings of Meeren et al. (2002) (Figure 1). They obtained local field potential recordings from a cortical grid covering the somatosensory cortex. In other experiments also the lateral dorsal (LD), ventral-postero-lateral (VPL) and ventral-postero-medial (VPM) thalamic nuclei were recorded. The model that was used were freely moving WAG/Rij rats, one of the well validated animal model of absence epilepsy (Depaulis and van Luijtelaar, 2006). Meeren et al. discovered with the aid of a non-linear association analysis (Lopes Da Silva et al., 1989; Pijn et al., 1990) that SWD always started locally, more specifically in the perioral-somatosensory cortex whereas all other cortical and thalamic recordings lagged behind. During the first 500 ms of SWD this focal region was found to drive other cortical and thalamic sites, whereas thereafter cortex and thalamus turned into an alternating resonance state (with cortex and thalamus taking turn in driving each other) (Meeren et al., 2002; Stefan and Lopes Da Silva, 2013).

Meeren et al. proposed that the perioral somatosensory cortex containes a “hot spot” that initiates a cascade of events that ultimately lead to the occurrence of the bilateral and generalized SWD, if the thalamo-cortical circuitry is in an appropriate state (Meeren et al., 2002, 2005; van Luijtelaar and Sitnikova, 2006). In this theory, a much stronger role is attributed to the cortex since both the role of the rhythm generator as well as hyperexcitable initiator of SWD is attributed to this local region. The thalamus is regarded as a relevant resonator for the maintenance of the SWD.

This strong contribution of the cortex as the origin was further strengthened by Polack et al. (2007), who confirmed the cortical focus theory by showing that neurons in the deep layers (V and VI) of the somatosensory cortex in GAERS, another well validated absence model (Marescaux et al., 1992; Depaulis and van Luijtelaar, 2006), show an increase in firing rate shortly before SWD onset, while this was not the case in the upper layers of the somatosensory cortex as well as in the motor cortex.

Whereas some inconsistent experimental results accumulated in the past years for the cortico-reticular theory (Pinault and O'Brien, 2005; van Luijtelaar and Sitnikova, 2006; Leresche et al., 2012), the cortical focus theory seems now in the spotlight of attention (van Luijtelaar et al., 2011b). Nevertheless, this theory is only a beginning of a description (a new idea) on how the cortico-thalamo-cortical system is involved in the generation of SWD, while several questions remain unanswered. This review summarizes recent data from mostly network analytical studies, which evaluate and extend the cortical focus theory. It will give a description of some aspect of “the cascade of events” that ultimately leads to the generation of full blown, bilaterally synchronized SWD (Section A Cascade of Events: Dynamics of Cortico-Thalamo-Cortical Network Interactions); and it will disentangle different functional contributions between cortex and different thalamic nuclei, relevant for the generation, maintenance and termination of SWD (Section Different Functional Contributions of Network Structures for SWD Generation, Maintenance and Termination). While Sections A Cascade of Events: Dynamics of Cortico-Thalamo-Cortical Network Interactions and Different Functional Contributions of Network Structures for SWD Generation, Maintenance and Termination are based on data as obtained in genetic rodent models of absence epilepsy, Section Imaging Studies in Children with Childhood Absence Epilepsy will give an overview on network activity in humans with absences, outlining and discussing similarities and differences with the animals with absences. An overall discussion and conclusion will then be given in Section Epilog: SWD – More Than A Cortical Focus.

## A cascade of events: dynamics of cortico-thalamo-cortical network interactions

**Figure 2:**
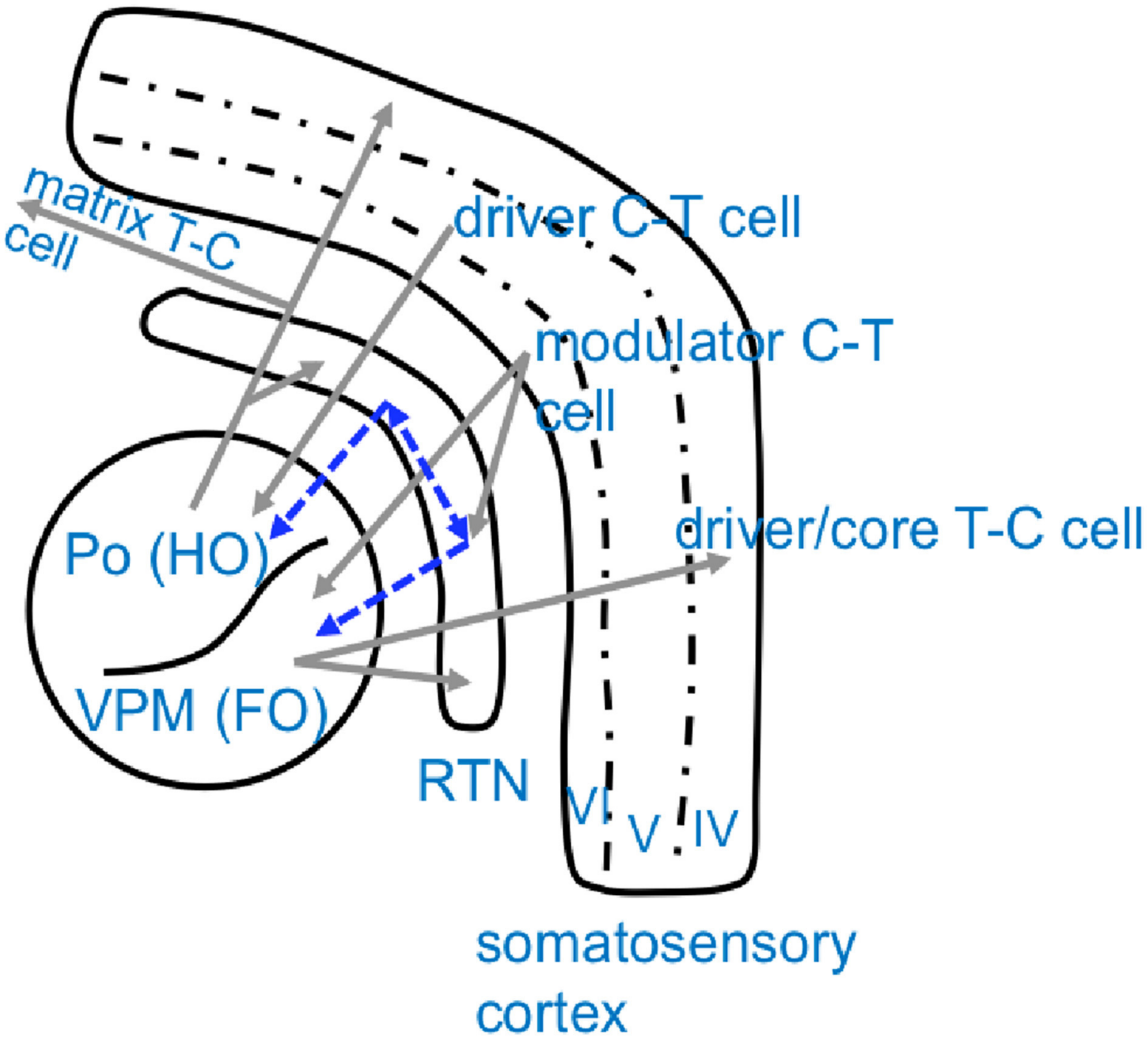
**Schematical overview of anatomical connectivity of the somatosensory cortico-thalamic loop**. Excitatory, glutamatergic connections are displayed in gray while inhibitory, GABAergic projections are displayed in blue. Note that thalamic nuclei are chategorized as either higher order nuclei (HO), which receive their driving input from the cortex and project widespreadly to other cortical regions, and first order nuclei (FO), which receive their driving input from sensory organs and project to a defined/restricted cortical area (Sherman and Guillery, 2005). Po, Posterior thalamic nucleus; VPM, Venral-Postero-Medial thalamic nucleus; RTN, Reticular thalamic nucleus; C-T cell, cortico-thalamic cell; T-C cell, thalamo-cortical cell.

Dynamics of network interactions which ultimately lead to the generation or termination of “full blown” SWD were investigated in a series of signal analytical studies by Lüttjohann and colleagues in WAG/Rij rats (Lüttjohann and van Luijtelaar, 2012; Lüttjohann et al., 2013, 2014). They acquired local field potential recordings in an extended part of the cortico-thalamo-cortical system. Cortical electrodes were positioned at the deep layers of the somatosensory cortex (ctx5 and ctx6), the proposed location of the epileptic focus in GAERS and WAG/Rij rats (Meeren et al., 2002; Polack et al., 2007; van Luijtelaar et al., 2011b), and in layer IV of the somatosensory cortex (ctx4), the major input layer from the thalamus. Thalamic electrodes were located at the ventro-postero-medial thalamic nucleus (VPM), receiving projections from layer VI and projecting to layer IV of the somatosensory cortex, at the posterior thalamic nucleus (Po), receiving projections from layer V and projecting widespread to the somatosensory cortex and other cortical areas, at the caudal and rostral Reticular thalamic nucleus (cRTN and rRTN), as potential synchronizers of the system, as well as the anterior thalamic nucleus (ATN) interconnected with the rostral RTN (Lu and Lin, 1993; Bourassa et al., 1995; Gonzalo-Ruiz and Lieberman, 1995; Deschênes et al., 1998; Sherman and Guillery, 1998, 2005; Veinante et al., 2000; Killackey and Sherman, 2003; Oda et al., 2004; Huguenard and McCormick, 2007) (For a schematic overview of the anatomical pathways see Figure 2).

Network interactions between these structures were assessed along pre-SWD->SWD and SWD->post SWD transition periods, centered either around the first epileptic spike simultaneously present in cortex and thalamus (FCTS) or the last epileptic spike simultaneously present in cortex and thalamus (LCTS) respectively. Signals were analyzed with the aid of different, partly complementary, methods such as non-linear association analysis, it estimates the degree and direction of linear and non-linear coupling (Lopes Da Silva et al., 1989; Pijn et al., 1990), Pairwise Phase Consistency analysis (PPC), it assesses linear phasic relationships between signals (Vinck et al., 2010) and linear, frequency resolved, non-parametric Granger Causality analysis (Dhamala et al., 2008), another direct connectivity analysis. Coupling within the transition periods was compared to coupling during non-epileptic passive wakefulness control periods, a vigilance state during which a major proportion of SWD emerges (Drinkenburg et al., 1991). In addition, SWD->post SWD transitions were also compared to so called “stable SWD” periods, acquired from the middle of SWD.

**Figure 3:**
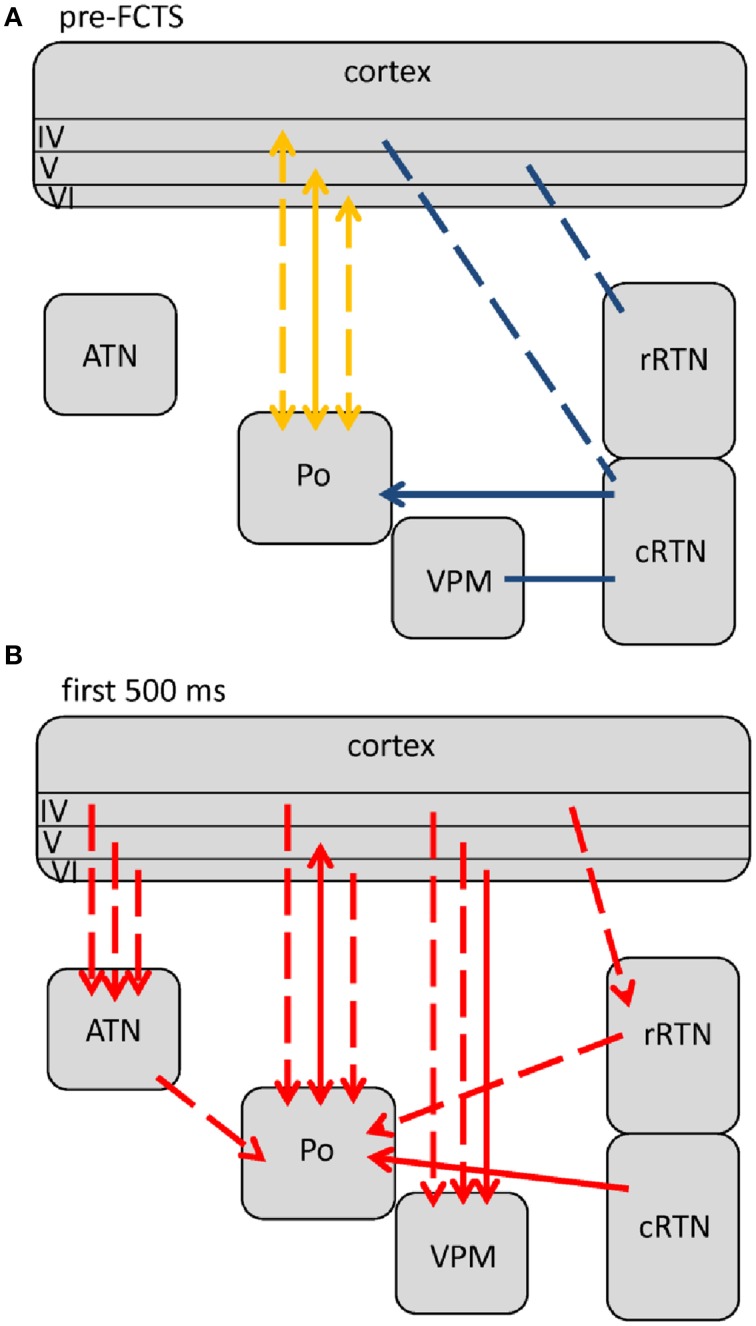
**Changes (as compared to non-epileptic control) in network interactions seen with SWD generation**. All pre-ictal changes are represented in **(A)**. Earliest pre-ictal changes were detected up 1.25 s prior to SWD onset or better the FCTS (first cortico-thalamic spike of the SWD). **(B)** Represents network coupling seen within the first 500 ms following FCTS. Solid lines represent changes between anatomically connected structures, dashed lines changes between structures that do not possess a direct, anatomical connection. The direction of coupling is indicated by arrowheads, which can either be unidirectional (→) or bidirectional (↔). Orange indicates a significant increase that did not yet reach its maximal value at that time point, red indicates that the coupling reached its maximal value and blue indicates a decrease in coupling.

**Figure 4:**
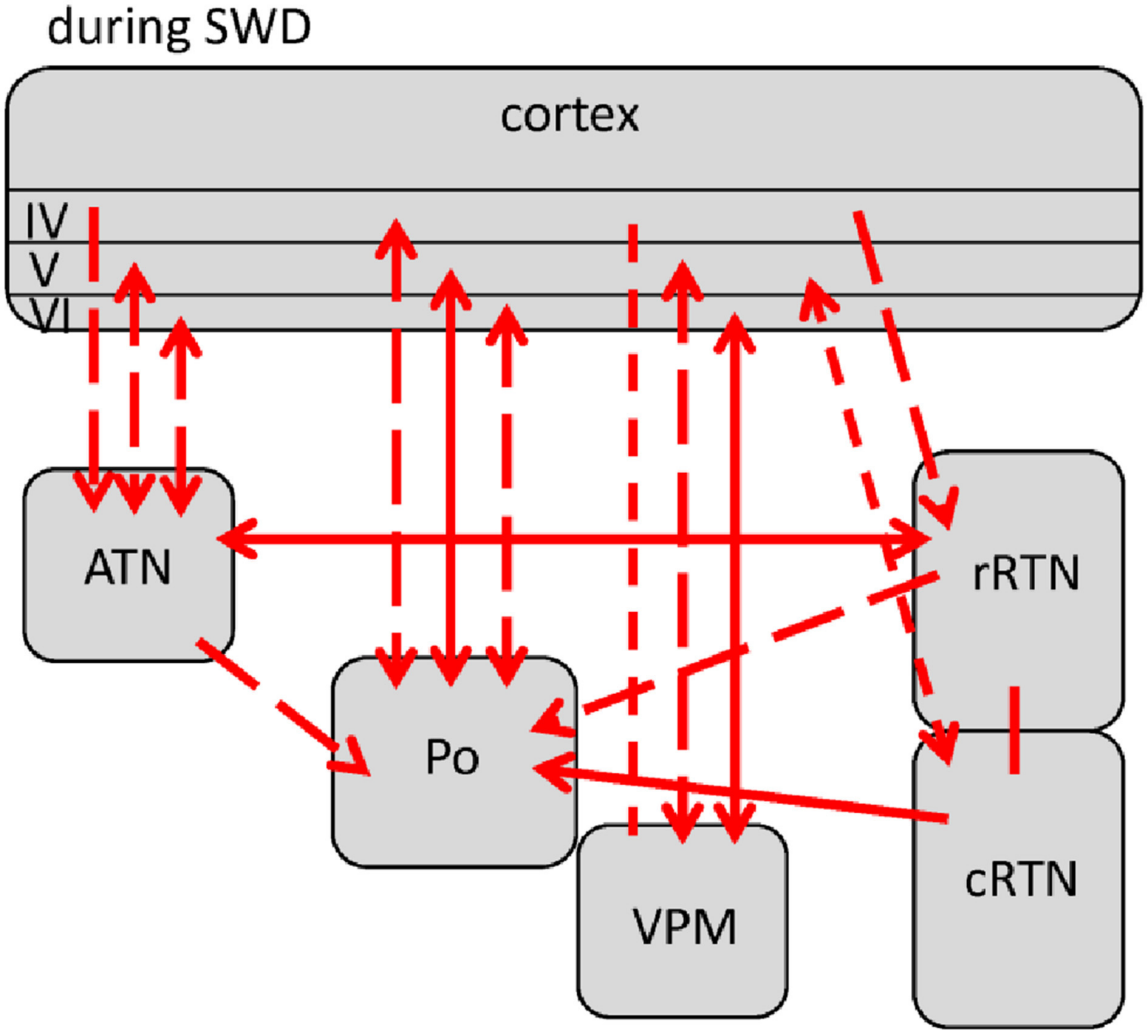
**Changes (as compared to non-epileptic control) in network interactions seen with SWD maintenance**. These are detected following the first 500 ms after SWD onset until 3 s following FCTS (= end of analysis interval) as well as 3 to 1.5 s prior to SWD termination or better the LCTS (last cortico-thalamic spike of the SWD). Solid lines represent changes between anatomically connected structures, dashed lines changes between structures that do not possess a direct, anatomical connection. The direction of coupling is indicated by arrowheads, which can either be unidirectional (→) or bidirectional (↔). Red indicates a significant increase that is at its maximal value.

**Figure 5:**
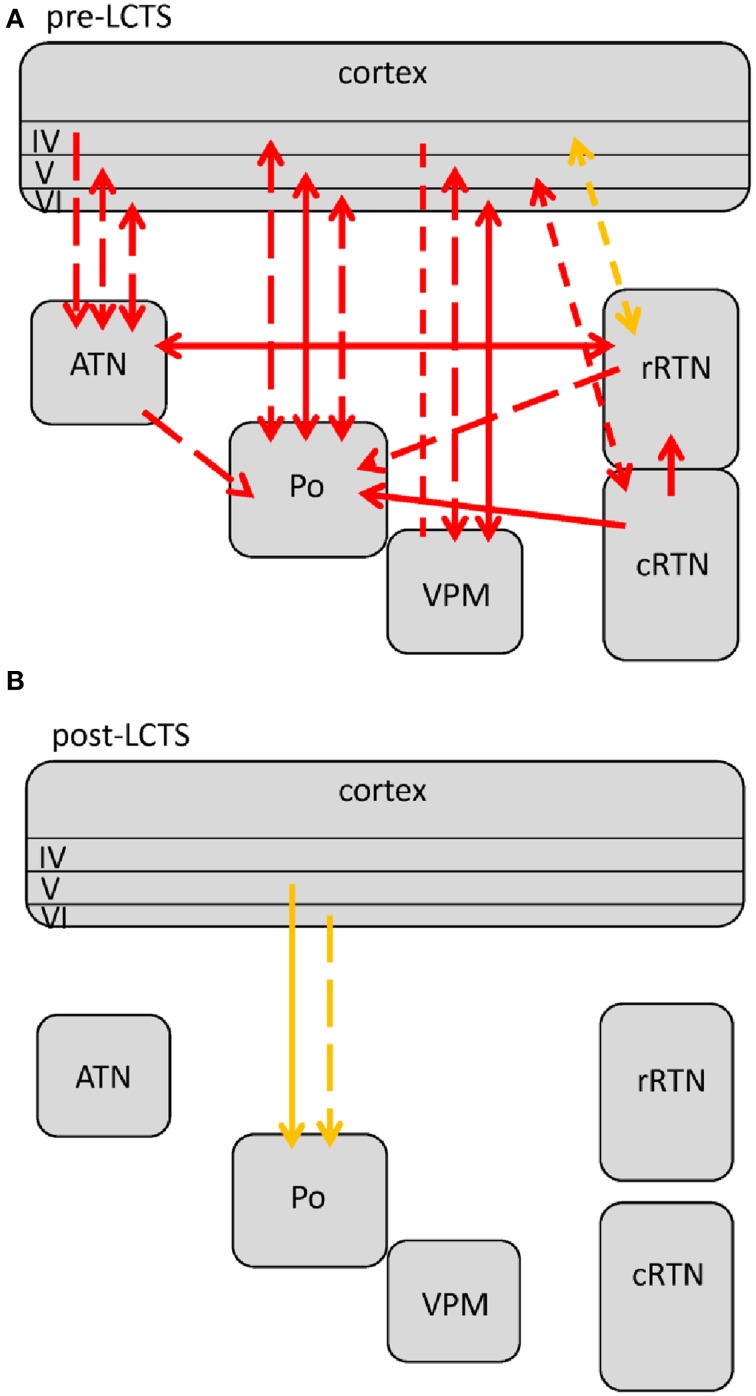
**Changes in network interactions seen with SWD termination**. **(A)** Represents changes seen from about 1.5 s prior to the LTCS (last cortico-thalamic spike of the SWD) until LCTS. **(B)** Represents changes from LCTS until about 1.5 s following it. Solid lines represent changes between anatomically connected structures, dashed lines changes between structures that do not possess a direct, anatomical connection. The direction of coupling is indicated by arrowheads, which can either be unidirectional (→) or bidirectional (↔). Orange indicates a significant increase that is not at its maximal value at that time point, red indicates that the coupling is at its maximal value.

Comparisons revealed 5 different profiles of network connectivity covering the pre-ictal to the post-ictal state. They are displayed in Figures 3A,B, 4, 5A,B. Significant relative changes in network interactions are either indicated as solid lines for changes between anatomically connected structures or as dashed lines for structures that do not possess a direct, anatomical connection. The direction of coupling is indicated by arrowheads, which can either be unidirectional (->) or bidirectional (<->). Since no directionality can be inferred from PPC analyses, a line without arrowhead is used if the direction of coupling cannot be inferred from one of the other “directed” methods. The strength of connectivity changes is color coded with orange indicating a significant increase that did not yet reach its maximal value at that time point compared to non-epileptic control periods, red indicates that the coupling reaches its maximal value and blue indicating a decrease in coupling, again in comparison with non-epileptic control periods. Figures 3–5 represent a summary of network changes detected in three different network analytical studies in which three different signal analytical methods were used. Only relative changes are displayed. The reader is referred to the original articles for the absolute changes (Lüttjohann and van Luijtelaar, 2012; Lüttjohann et al., 2013, 2014).

The five connectivity profiles are grouped into 3 consecutive figures, characterizing “network” processes of SWD generation, maintenance and termination, respectively.

### A scenario of SWD generation

Earliest changes in connectivity related to the generation of SWD could be detected more than a second prior to FCTS (Figure 3A):

At this state, the deep layers of the somatosensory cortex and the strong, reciprocally connected posterior thalamic nucleus, started to gradually increase their communication in a bidirectional fashion, until this reached a maximum around FCTS. This early increase was detected by non-linear association analysis (Lüttjohann and van Luijtelaar, 2012), not by PPC and GC. Therefore, it is likely that this increase in communication is non-linear, since it cannot be detected by linear connectivity methods like PPC or GC. This suggestion is supported by outcomes from a study of Sysoeva et al. (2014): these authors compared the sensitivity of a non-linear and linear Granger Causality analysis applied to SWD recorded in the frontal cortex and VPM. They reported that the non-linear variant was more sensitive to detect early (pre-ictal) changes between cortex and thalamus at SWD onset, while the linear version of GC failed to detect those. The cortico-thalamic coupling, as described by Meeren et al. (2002) was also non-linear, while cortico-cortical coupling increases seen during SWD were linear. Indeed epileptic seizures and possibly their pre-ictal preparation processes are often regarded to be of non-linear nature. Signal analyses derived from the theory/mathematics of non-linear dynamical systems are proposed to be of particular value for the understanding of seizure generation mechanism, potential preictal seizure preparation phases, as well as to possibilities of seizure prediction (Lehnertz et al., 1999; Le Van Quyen et al., 1999; Litt and Echauz, 2002; Lopes Da Silva et al., 2003a,b; Stefan and Lopes Da Silva, 2013). In line with this, non-linear changes have been reported for a different kind of seizure: Martinerie et al. (1998) reported changes in non-linear coupling 2–6 min before seizure onset between amygdala and posterior hippocampus in patients with temporal lobe epilepsy.

Changes in linear coupling were detected pre-ictally, too (Lüttjohann et al., 2013): a linear, phasic decoupling between the cRTN and layer IV of the somatosensory cortex and between the rRTN and layer V was observed, which was shortly followed by a phasic decoupling between cRTN and Po. Thereafter, still about 1 s prior to FCTS, a decoupling from cRTN to VPM was noticed. It is important that in these pairs coupling decreased. The decoupling between cRTN and VPM may be regarded as an argument not fitting the cortico-reticular theory, which assumes that sleep spindles are the constituent oscillation of SWD. Sleep spindle generation, however, requires an increased influence of the RTN onto thalamo-cortical relay cells (Steriade, 2003), which is at odds with a decoupling between cRTN and VPM found in the current study.

As it has been proposed for convulsive seizures in Temporal-Lobe-Epilepsy (TLE) (Martinerie et al., 1998), preictal changes in network activity might also pave the way for SWD prediction. Such a prediction however, would require a certain sensitivity (they should be present before all SWD) and specificity (they should preferably be present only before the SWD) of the pre-ictal changes.

Earlier, Sitnikova and van Luijtelaar (2009), invested power spectra of pre-SWD epochs, recorded in the frontal and occipital cortex as well as in the VPM and rRTN of WAG/Rij rats. Four distinct power spectra profiles were described in the 1 s epoch immediately preceding the onset of SWD. Comparison of changes in coherence calculated for the 1 s pre-SWD and the first second after SWD onset, in the same study, however, revealed no unique coupling profile for SWD with different pre-SWD power spectra. Rather different profiles in coupling changes were revealed for intracortical, intrathalamic, and cortico-thalamic channelpairs, respectively. This demonstrates that the size of the coupling change depends on the channelpair that is analyzed. Similar selective changes in coupling were found preictally (Lüttjohann and van Luijtelaar, 2012; Lüttjohann et al., 2013).

van Luijtelaar et al. (2011a) applied a wavelet analysis to the same data-set (frontal cortex and VPM) as Sitnikova, they reported the existence of delta-theta precursors in cortex and thalamus which were present in 80–90% of pre-SWD epochs and which were virtually absent in other randomly selected vigilance states. Preictal delta-theta power was also found by us (Lüttjohann et al., 2013).

Together, it can be concluded safely that in contrast to the long lasting view that SWD are sudden and unpredictable events (ILAE, 1981), SWD occurrence as studied in genetic absence models is a gradual process which already starts more than a second prior to the onset of a full-blown SWD. Such early changes in network activity might also open the possibility for SWD prediction.

At FCTS, the decreased coupling values of the pre-ictal epoch returned to normal baseline values, while the increased coupling between cortex and Po remained present, see Figure 3B. At this time point, additional increases in coupling strength between the deep layers of the cortex and VPM, ATN, rRTN could be seen. In line with the cortical focus theory is that in all these cases coupling increases were of unidirectional nature with the cortex driving the thalamus. GC revealed that the drive for the anatomically connected ctx6-VPM occurred in the 4–10 Hz range, while the drive between ctx6-ATN occurred in the 40–50 Hz.

Channelpair ctx5-Po was an exception in the sense that it was the only pair that kept a bidirectional crosstalk reaching a maximum increase at about 375 ms following FCTS. Moreover, this channelpair had higher PPC values than any other pair. (See Section Different Functional Contributions of Network Structures for SWD Generation, Maintenance and Termination for interpretation).

PPC analysis for this pair revealed an increase in a broad (8–48 Hz) frequency range, while for the other cortico-thalamic pairs, noted above, PPC increases were seen in the 8–12 Hz band and in its harmonics.

In addition, unidirectional increases in thalamo-thalamic coupling also occurred at FCTS. Except for the VPM (for which inconsistent results were revealed by the different analysis methods), all thalamic nuclei (ATN, cRTN, and rRTN) consistently started to drive the Po. This drive, as well as increases in PPC, was found in the 4–12 Hz band (Lüttjohann et al., 2013).

### SWD maintenance

About 500 ms following FCTS the cortico-thalamo-cortical system proceeded into the third, more stable (i.e., longer lasting) coupling profile (Figure 4), which characterizes SWD maintenance.

During this phase all intra-thalamic coupling connections described in the “first 500 ms” coupling period remained the same. An additional increase of coupling was observed between cRTN–rRTN, ATN-rRTN, and ctx5-cRTN. In line with the predictions of the cortical focus theory (the cortical focus drives the thalamus during the first 500 ms), most cortico-thalamic connections turned into a bidirectional crosstalk, with cortex and thalamus taking turns in driving each other. However, there were two exceptions: channelpairs ctx4-rRTN and ctx4-ATN; here the cortical drive was maintained. As was the case for the bidirectional crosstalk between ctx5-Po seen during the first 500 ms, this observation extends the cortical focus theory by showing that the thalamus is not homogeneous in its interactions with the cortical focus (for more details see Section Different Functional Contributions of Network Structures for SWD Generation, Maintenance and Termination). Whereas for channelpair ctx4->ATN this drive was found in higher frequencies (20–50 Hz), the driving force between ctx4->rRTN was found in the SWD characteristic 8–12 Hz band and in the 20–50 Hz band.

### A scenario of SWD termination

Visual inspection around the offset of SWD already revealed differences in the time point of SWD termination between cortex and thalamus. In about 20% of rats, the thalamic SWD activity was found to stop about 1 s earlier than cortical SWD activity.

Two changes were noticed within the pre-LCTS period as compared to the SWD maintenance period (compare Figures 4, 5A) with respect to coupling dynamics: First, the directional drive from ctx4 onto -> rRTN found for the SWD relevant 8–12 Hz band in the SWD maintenance coupling profile, diminished at about 1.5 s prior to LCTS. Second, an increase in the directional drive between cRTN driving (->) the rRTN was observed about 1 s prior to LCTS. This was followed (Figure 5B) by a return to baseline coupling values seen for all except two channelpairs (ctx5-Po and ctx6-Po) at FCTS. Only ctx5-Po and ctx6-Po kept their increased coupling strength for another 1.5 s following FCTS. In contrast to the SWD maintenance and the (ctx5-Po) SWD initiation period, the increased coupling between cortex and Po was not bidirectional, but unilateral with the cortex driving the Po (Figure 5B) (see Section Different Functional Contributions of Network Structures for SWD Generation, Maintenance and Termination for Interpretation).

Given the early changes found in the pre-LCTS period (decrease in directional drive ctx4-rRTN 1.5 s prior to LCTS and increased directional drive cRTN-rRTN), which seem to initiate the massive coupling loss at LCTS, it can be concluded that comparable to SWD generation, SWD termination is also a gradual rather than an abrupt process. Sitnikova et al. (2008) also reported a pre-LCTS reduction in the strength of frontal cortex—VPM coupling, suggesting that other cortical regions also reduce their communication to the thalamus prior to SWD offset.

It can be concluded that both cortico-thalamic as well as intra-thalamic processes participate in this initiation of SWD termination. However, both the cortico-thalamic and the intra-thalamic coupling changes associated with SWD termination include the rRTN. In contrast to the network analysis on SWD termination, network analysis on sleep spindle termination (Timofeev et al., 2001), demonstrated a major role of, predominantly, the cortex-i.e the cortical cells were found to start firing out of phase and a computer model, lacking the cortico-thalamic input, was found to allow continuous spindle oscillations within the thalamus RTN network. This might indicate that sleep spindles and SWD are fundamentally different types of oscillations.

It is proposed that the above outlined changes might be prerequisites for either the generation or termination of bilaterally synchronized SWD (See Section Different Functional Contributions of Network Structures for SWD Generation, Maintenance and Termination for an interpretation on the relevance of the above described changes for SWD initiation or termination, respectively).

## Different functional contributions of network structures for SWD generation, maintenance and termination

In the following section, different functional roles are proposed for the cortex and the different thalamic nuclei in SWD occurrence in the genetic rodent models.

### The somatosensory cortex: three characteristics of an epileptic focus

The cortical focus theory assumes that the deep somatosensory cortex contains hyperexcitable cells, forming a focal region, in which SWD are initiated, while the thalamus is regarded as a necessary resonator for the full-blown SWD to occur and maintain.

For this theory to be true, the focal region in the deep somatosensory cortex requires three characteristics: (a) it should drive other structures; (b) it should have local rhythm generating abilities and (c) it should show locally enhanced excitability.

#### Driving forces onto other structures

This first prerequisite has been validated by the original study of Meeren et al. (2002): they showed that the perioral region of the somatosensory cortex of the absence epileptic WAG/Rij rat drives other parts of the cortex and the ventral basal complex of the thalamus within the first 500 ms of the SWD. We conformed and extended their original finding by more extensive analysis of intrathalamic, cortico-thalamic, and thalamo-cortical interactions. These studies, as described in the previous section, showed the same driving capacities of the somatosensory cortex for the various (except one, see below) thalamic nuclei.

On the other hand, it needs to be mentioned that electrophysiologic studies *per se* include a limited spatial resolution so that theoretically there could always be a third “unrecorded” structure, which can explain the demonstrated results, since this structure may drive both the somatosensory cortex as well as the thalamic nuclei with different temporal delays.

While Lüttjohann et al. only showed that the deep somatosensory cortex drives most thalamic nuclei, they did not exclude that also other cortical structures drive thalamic nuclei. On the other hand, Sitnikova et al. (2008) demonstrated with the aid of linear GC that the motor cortex did not drive the VPM at the onset of SWD.

A more extended study of David et al. (2008) performed a Granger causality on high spatial resolution fMRI data in the GAERS model. These authors defined regions of interest for the determination of coupling dynamics, including primary somatosensory cortex (barrel field), primary somatosensory cortex (limb region), secondary somatosensory cortex, secondary motor cortex, visual cortex, retrosplenial cortex, thalamus, striatum, substantia nigra, pons, cerebellum and medulla oblongata based on changes in the BOLD signal seen with SWD occurrence. After a hemodynamic deconvolution it was concluded that the somatosensory cortex drives other brain areas. This fMRI study minimizes the risk that there might be a third unrecorded structure next to the somatosensory cortex, so that it can safely be concluded that the somatosensory cortex of absence epileptic rats possesses driving forces onto both other cortical and thalamic structures at SWD onset. This fulfills the first prerequisite of an epileptic focus.

#### Local rhythm generating abilities

Visual inspection (i.e., without the application of a signal analytical method) and quantification of the SWD in our recordings obtained revealed that in about 20% of rats, SWD activity can be seen to start up to 1 s earlier in the deep somatosensory cortex as compared to all other thalamic nuclei. In addition, in about 20% of the rats local SWD-like activity could be found, which was only present in the deep layers (IV-VI) of the somatosensory cortex, while the thalamic recordings were unaltered (van Luijtelaar et al., 2011b; Lüttjohann and van Luijtelaar, 2012). The absence of these observations in other rats might be explained by an inter-individual variability in the location of the focus (Meeren et al., 2002).

Recordings of local thalamic SWD activity or SWD activity that can be seen, on visual inspection, to start earlier in the thalamus than in the deep layers of the somatosensory cortex, were never encountered. So called “embryonic SWD,” reminiscent to the local SWD were also reported by Seidenbecher et al. (1998) in anesthetized GAERS rats. These “embryonic SWD” were recorded concomitant to SWD correlated firing in layer IV and V of the somatosensory cortex, which precede the main, generalized, cortico-thalamo-cortical SWD (Seidenbecher et al., 1998). In GAERS, SWD are reported to arise out of 5–9 Hz medium voltage oscillations, reminiscent to the sensory-motor-rhythm (Pinault et al., 2001, 2006), another cortically generated rhythm (Nicolelis et al., 1995). In a recent study by Zheng et al. (2012) these oscillations were related to local increases in interactions between primary and secondary somatosensory cortex as well as the adjacent insular cortex, highlighting the importance of S2 and insular cortex as primary SWD onset zone in GAERS. Indeed, several studies pointed to a slight difference between GAERS and WAG/Rij rats regarding the location of the cortical, epileptic focus, with S2 as location of the focus in GAERS (Pinault et al., 2001, 2006; Gurbanova et al., 2006; Polack et al., 2007, 2009; Zheng et al., 2012) and S1_perioral_ as location of the focus in WAG/Rij rats (For a detail review on similarities and differences between both genetic strains see Akman et al. (2010). Whether also in WAG/Rij rats there is a somewhat broader local cortical initiation network (e.g., S1, S2, insular cortex instead of just S1) and whether this is also the primary network for the pre-SWD activity in WAG/Rij rats, remains to be investigated.

On top of that, local, low intensity (range 20–100 μA), electrical stimulation of the deep somatosensory cortex of WAG/Rij rats, but not (or to a much lesser extent) of healthy Wistar control rats, induced rhythmic, self-sustained afterdischarges (AD) lasting several seconds, which strongly resemble the spontaneous occurring SWD (Lüttjohann et al., 2011). This yields functional evidence that the somatosensory cortex of WAG/Rij but not of Wistar rats is able to produce SWD (type of oscillations). The presence of stimulation induced AD-SWD was confirmed in GAERS (Zheng et al., 2012). Stimulation of the motor cortex was also able to induce AD-SWDs in WAG/Rij rats. However, these were shorter and had a less stable 8 Hz rhythm than AD elicited in the somatosensory cortex. This might either be due to excitability differences of the cortical areas or differences in the somatosensory circuit vs. motor circuit (Lüttjohann et al., 2011). A recent modeling study demonstrated that heterogeneity of cortical tissue can explain differences in AD properties (Goodfellow et al., 2012). The circuit specificity for SWD like ADs is in agreement with outcomes from a thalamic stimulation study: stimulation of the VPM, which is part of the somatosensory loop, could easily induce AD, while stimulation of the ATN, a nucleus that belongs to the limbic system, only rarely induced AD (Lüttjohann and van Luijtelaar, 2013). The anatomical connections of the focal region with the motor cortex and VPM, but not ATN, allows the possibility that in the case of motor cortex and VPM stimulation AD induction might be the result of activation of the epileptic focus in the somatosensory cortex.

These results favor the conclusion that the somatosensory cortex also possesses the second prerequisite of an epileptic focus, namely local rhythm generating abilities.

#### Locally enhanced excitability

The most direct experimental test of the existence of a hyperexcitable spot that can function as initiator of SWD *in vivo*, in un-anesthetized rats was performed in an electrical stimulation study (Lüttjohann et al., 2011). The amplitude of electrically elicited and recorded evoked potentials (EEP) in either the deep somatosensory cortex or the adjacent motor cortex in WAG/Rij rats and Wistar control rats was significantly increased only in the deep somatosensory cortex of WAG/Rij rats. Since early components of an evoked potential are known to be mainly dependent on stimulus properties while later components can also be influenced by network and top-down processes (Coenen, 1995), the local selective increase of the N1 amplitude in the epileptic rat strain is an excellent biomarker for the selective increase in local excitability of the deep somatosensory cortex of these rats. The increase of the late N3 component, on the other hand, might indicate an additional heightened excitability of the network. The increase in N1 EEP amplitude was independent of the level of alertness (wakefulness, drowsiness and deep slow wave sleep), showing that the enhanced excitability is a “permanent” characteristic of the deep somatosensory cortex of adult (older than 6 months) WAG/Rij rats. As a consequence, the underlying cause of this higher excitability needs to be attributed to a “permanent” factor.

The nature of this increased excitability can most likely be attributed to the sum of local changes promoting excitation in itself as well as factors that lead to an increased excitation by inducing a local reduction of inhibition. As reviewed by van Luijtelaar et al. (2011b), several changes were found in the somatosensory cortex of absence epileptic rats, including an upregulation of particular subtypes of Na^+^ channels (Klein et al., 2004), increased NMDA receptor-mediated activity (Pumain et al., 1992; Luhmann et al., 1995; D'antuono et al., 2006), a decrease in the efficacy of GABA_A_ or GABA_B_-ergic inhibition (Merlo et al., 2007; Inaba et al., 2009) and an increased expression of mGlu2/3 receptors (Ngomba et al., 2005). Furthermore, less NMDA-NR1 and AMPA-GluR4 subunits were reported in the subgranular layers, not directly in line with evidence for an excitable region, unless this implies a decrease in the strength that cortical GABA-interneurons are stimulated or as a consequence of any other change in biochemical parameters (van de Bovenkamp-Janssen et al., 2006).

One of the most elegant documented changes in the somatosensory cortex of WAG/Rij rats is a reduction in hyperpolarization-activated cation current (I*h*) (Strauss et al., 2004; Schridde et al., 2006; Kole et al., 2007). This reduction of HCN1 channels was also age related, and in parallel with the age dependent increase in SWD. However, the time-course of the receptor decrease preceded the time-course of SWD ontogeny (Kole et al., 2007). Therefore, it seems that the decrease in HCN1 cannot be caused by the exposure of the brain to hundreds of SWD per day, but might rather be the result of a genetic predisposition toward a diminishment of HCN1 channels. This in turn makes the rats more prone to develop SWD during ontogeny. Next to this, Blumenfeld and colleagues demonstrated that an early onset of a long-term treatment with ethosuximide prevented or delayed both the ontogeny of SWD as well as the HCN1 channel loss within the somatosensory cortex of WAG/Rij rats (Blumenfeld et al., 2008), indicating a causal role between HCN1 channel loss, resulting in enhanced excitability of the somatosensory cortex as well as the development of SWDs during ontogeny. Interestingly, such a long term treatment with ethosuximide was also shown to influence locally evoked and recorded EEPs: WAG/Rij rats showed a higher inhibitory component of the EEP compared to age-matched untreated animals. Furthermore, long-term ethosuximide treatment decreased the number and duration of stimulation induced ADs (regarded as the indicator of epileptic rhythm generating properties of the somatosensory cortex, mentioned above) compared to untreated rats (van Luijtelaar et al., 2013).

### The posterior thalamic nucleus: a “reverberator”

The Posterior nucleus of the thalamus possesses strong reciprocal connections to layer V of the somatosensory cortex (Deschênes et al., 1994). Although earlier invasive and non-invasive studies indicated its participation in SWD occurrence (Vergnes et al., 1987; Tenney et al., 2004), the Po has received little to no attention in previous studies on SWD generation and has not been included in any computational model of SWD generation. Most studies predominantly focused on the role of the VPM and RTN and only a few researchers proposed, in theoretical scenarios, that the Po with its widespread connections to other cortical areas might help to spread and generalize SWD activity (Kostopoulos, 2001; Polack et al., 2007, 2009).

The Po showed the earliest pre-ictal, as well as one of the strongest ictal, increases in coupling to the epileptic focus. This characteristic makes it an interesting candidate for the initiation of “full-blown” SWD. It could not be excluded until now that part of these early increases in connectivity might be attributable to changes in the level of vigilance (from awake to drowsiness and light slow wave sleep). However, the Po is also characterized by a third behavioral characteristic, which makes it highly likely to be of crucial relevance for SWD generation: it is the only nucleus, recorded in this study, which kept a bidirectional crosstalk to the epileptic focus during the first 500 ms of SWD. In other words, the Po is the only thalamic nucleus which “responded” or gives feedback to the cortical epileptic focus within the early SWD generation stage. The importance of such a “responder” is expressed in a seminal review (Huguenard and McCormick, 2007). After describing the ideas of the cortical focus theory these authors state:

“This model would require that the neocortical network be receptive to the feedback excitatory TC activity, that is, that it not produce destructive interference that would destabilize the overall development of a global oscillation. This hypothesis remains to be tested.” (Huguenard and McCormick, 2007, Trends in Neurosciences, 30(7), p. 354).

**Figure 6:**
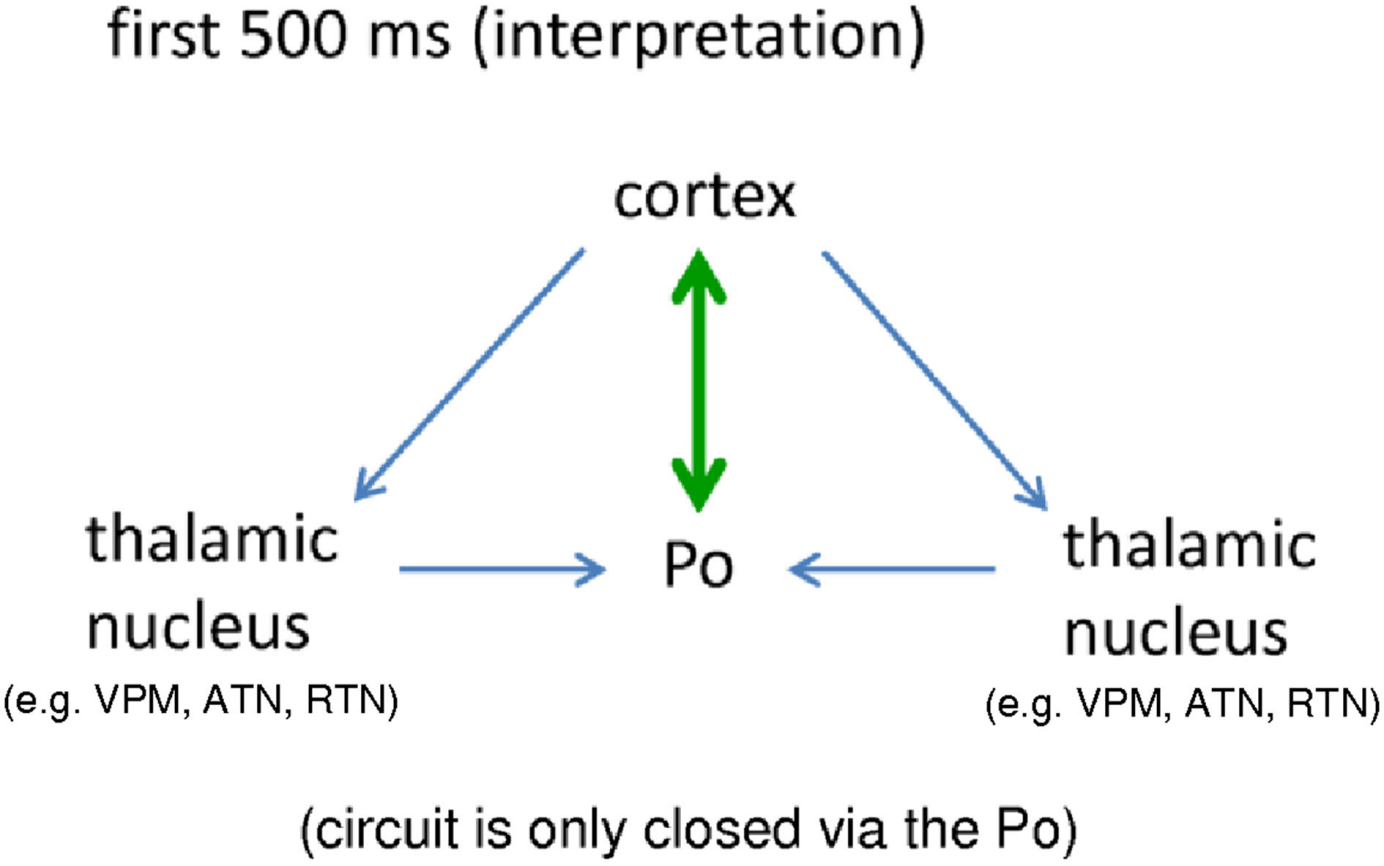
**Schematic interpretation of the importance of a bidirectional crosstalk between cortical, epileptic focus and posterior thalamic nucleus for the generation of “full blown” SWD (see text for details)**. Po, Posterior thalamic nucleus; VPM, Venral-Postero-Medial thalamic nucleus; RTN, Reticular thalamic nucleus; ATN, Anterior thalamic nucleus.

The strong bidirectional coupling between cortex and Po fulfills this prerequisite. In other words, a seizure generator (either in cortex or in thalamus) alone is not enough for the occurrence of bilateral, symmetrical and synchronized SWDs. Rather, an intact, closed cortico-thalamo-cortical loop, is required (see e.g., Avoli and Gloor, 1981; Sitnikova and van Luijtelaar, 2004). During the first 500 ms a closed loop of signal reverberation is only provided via the Po. It occurs either in the form of small, direct loop (Figure 6 green) established in the bidirectional ctx5<->Po connection, or, in a longer loop (Figure 6, blue) where the cortex sends activity to other thalamic nuclei, they send this activity to the Po and the Po returns the activity back to the cortex.

Interestingly, following the end of SWD, as described in Section A Cascade of Events: Dynamics of Cortico-Thalamo-Cortical Network Interactions, the cortex keeps an increased connectivity to the Po for another 1.5 s but at this stage the Po does not respond to the cortex anymore (Figure 5B).

Frequency modulation from about 11 Hz at the beginning of a SWD to about 6 Hz at the end of a SWD can be seen (Bosnyakova et al., 2006). Long lasting SWD may consist of several repetitions of frequency modulated short SWDs. At the end of such frequency modulated element, the SWD has a high chance to terminate, but continues or better, it restarts. Since the chance to get a new SWD is indeed highest following the end of SWD (Maris et al., 2006), this drive of the cortex to the Po might be interpreted as a SWD re-initiation attempt of the cortical focus. The prolonged local SWD activity in the deep somatosensory cortex after the thalamus no longer showed SWD, seen in about 20% of rats (van Luijtelaar et al., 2011b; Lüttjohann et al., 2014) might also favor the suggestion of a cortical drive to reinitiate SWD occurrence. It shows that the hyperexcitable cortex continues to produce localized SWD activity, but cannot generalize to thalamic structures anymore. The re-initiation attempt fails; the SWD activity cannot generalize to thalamic structures, since the Po does not respond to the cortex anymore. As a consequence, both the full-blown bilaterally generalized SWD stops immediately and even the local activity fades away due to a missing thalamic resonance circuit. It is also hypothesized that network analyses clustered around the endpoints of the repeated elements would show the same network changes as seen for the final termination, except for the fact that the Po keeps responding to the thalamus, so that the re-initiation or continuation of SWD is successful. Positive validation of this hypothesis in future network studies could confirm the important role of the Po for SWD generation and “re-initiation.”

It is not surprising from an anatomical point of view that the Po is the primary thalamic counterpart of the cortical focus. It is the higher order thalamic nucleus of the somatosensory system, which receives its main driving input from the somatosensory cortex via the so called “driver” cortico-thalamic cells, which act via fast ionotropic receptors (Sherman and Guillery, 1998). Indeed, the Po in GAERS contains a higher number of driver terminals, as compared to healthy non-epileptic control rats. This difference was not found in other thalamic nuclei (Cavdar et al., 2012). Furthermore, higher-order nuclei like the Po, as compared to first order nuclei such as the VPM, have been demonstrated to be more prone to thalamic burst firing (Ramcharan et al., 2005), a property which is generally believed to be favorable for SWD generation (Avoli et al., 2001).

Since the Po stays in its bidirectional crosstalk to the cortex, and continues to receive input from other thalamic nuclei during SWD maintenance, it might be possible that the Po is the major reverberator to the cortex. The Po gathers the intra-thalamic activity to generate a common synchronous feedback of feed forward signal to the cortex, which might support SWD maintenance. This, however, remains to be validated, since at this stage additional “closed loop circuits” are active (e.g., ctx<->VPM; see Figure 4), so that also other thalamic nuclei, next to the Po, can give feedback/ respond to the cortex and by this support the maintenance of the SWD.

Lastly, the Po might be involved in SWD generalization given its widespread cortical connections (Kostopoulos, 2001). So far no signal analytical studies were conducted in which coupling between the Po and multiple cortical areas were calculated. This implies that this hypothesis remains to be experimentally verified.

### The ventral-posterio-medial thalamic nucleus (VPM): a “trigger relay”

The VPM, in contrast to the Po, has always been regarded as belonging to the key-network of SWD generation (Danober et al., 1998; Seidenbecher et al., 1998; McCormick and Contreras, 2001; Coenen and van Luijtelaar, 2003; Meeren et al., 2005; Pinault and O'Brien, 2005; van Luijtelaar and Sitnikova, 2006; Huguenard and McCormick, 2007). This is probably a remainder of the cortico-reticular-theory, which assumes that SWDs are modified sleep spindles. Sleep spindles are generated in the intra-thalamic circuit, in which the GABAergic influence of the RTN hyperpolarizes TC cells and brings them into a burst firing mode. The VPM, as one of the major sensory relay cells was therefore, according to this theory, crucially involved in rhythm generation.

Results of the network analytical studies, however, indicate that the role of the VPM for SWD generation, as compared to the Po, might be smaller or at least different; In contrast to the Po, the VPM only showed late (earliest at FCTS) increases in coupling with the cortical focal area and these changes were only small to moderate. In addition, during the SWD maintenance phase, the VPM only showed consistent (verified by multiple connectivity methods) increases in coupling with the three layers of the somatosensory cortex, but, in contrast to the Po, not with other thalamic nuclei.

Given the fact that the VPM is a first order thalamic nucleus, or in the categorization schema of Jones a “core” cell that projects only to a localized region of the cortex (Jones, 2009), it is unlikely that the VPM is crucially involved in the generalization and maintainance of the SWD, which might, given the generalized nature of SWD, require a broader contact to the cortex. The Po, on the other hand, is a higher order nucleus that sends “matrix” cells to the cortex, i.e., it projects to multiple cortical areas.

In line with a more modest role of the VPM in SWD generation and maintenance, local application of the anti-absence drug ethosuximide to the VPM in GAERS was insufficient to block SWD completely (Richards et al., 2003). Furthermore, multiple unit recordings in the VPM in GAERS demonstrated that the VPM only seldom fires in a seizure-favorable burst-like fashion during SWD (Pinault and O'Brien, 2005; Leresche et al., 2012). Interestingly, Polack et al. (2009) demonstrated that the ratio of burst firing during SWD is higher in the Po as compared to the VPM. Likewise, direct comparison between Po and VPM revealed that strong increases in phase coupling between cortex and Po were seen in a much broader spectral domain compared to the VPM.

On the other hand, the VPM cannot be ignored in the process of SWD generation: Group I mGluR's, involved in SWD generation, are functionally deficient in symptomatic WAG/Rij rats (D'Amore et al., 2013). Next, Abbasova et al. (2010) demonstrated the importance of external (somato)-sensory input for the occurrence of SWD since functional inactivation of the peripheral trigeminal nerve almost completely abolished SWD. Given this importance of external input for the generation of SWD, the VPM gets a role in SWD generation from an anatomical and functional point of view. The VPM is a first order nucleus, which relays external stimuli, transported to the VPM by the trigeminal nerve, to layer IV of the somatosensory cortex. From layer IV intra-cortical connections (Thomson and Bannister, 2003) can transport the trigger input to the superficial cortical layers and to the deep layers (V and VI), which contain the epileptic focus. In line with this, h^2^ analysis showed a directional drive from layer IV to layer V and VI prior to FCTS (Lüttjohann and van Luijtelaar, 2012).

The function of a “trigger input relay” is therefore proposed for the VPM. This is supported by our finding that low frequency stimulation of the VPM induced SWD-like AD, whereas stimulation of the ATN, a nucleus, that does not relay information to the somatosensory cortex was much less sensitive and revealed much less AD (Lüttjohann and van Luijtelaar, 2013). Finally, functional deactivation of the VPM blocked the occurrence of SWDs in GAERS (Danober et al., 1998).

The somatosensory cortex is known to contain so-called “intrinsically bursting” cells (Connors and Gutnick, 1990), which react to incoming stimuli from the VPM with a stereotyped burst firing pattern. These cells are present in the primary and secondary somatosensory cortex, and their bursting contingent upon activity in the VPM may form the basis of a first local epileptic SWD of the somatosensory epileptic focus (Zheng et al., 2012).

### The reticular thalamic nucleus: more than one structure/function

The RTN contains different functional subparts. The rRTN is part of the limbic circuitry, the caudal part possesses different functional compartments involved in modulating sensory information transfer to the cortex (Pinault, 2004). However, this distinction between functional subparts was not always taken into account regarding its role in the pathophysiology of SWD. A study by Aker et al. (2006) and Meeren et al. (2009) pointed toward the importance of discriminating between the rostral and caudal parts of the RTN regarding SWD generation and maintenance. Both groups showed that local pharmacological inhibition or lesioning of the rostral RTN decreased SWD activity while the same treatment to the caudal part of the RTN resulted in an increase. Functional interactions during SWD generation, maintenance and termination, summarized in Section A Cascade of Events: Dynamics of Cortico-Thalamo-Cortical Network Interactions of this review, confirm the existence of functional subparts within the RTN and help to pinpoint its relevance for SWD.

#### The rostral reticular thalamic nucleus, a “resonator”

The rRTN was continuously guided by the somatosensory cortex (layer IV) in the high frequencies (20–50 Hz) and until 1.5 s prior to LCTS in the SWD typical 8–12 Hz band. While an 8–12 Hz firing rate is known to be the intrinsic firing rhythm of TC and CT cells (Golshani and Jones, 1999), the high frequency drive might be a result of active GABAergic neurons, which are known to be able to fire at a higher frequency. In line with this, changes (increases) in the spectral EEG content by GABAergic drugs are predominantly seen in this (20–50 Hz) frequency range (Coenen and van Luijtelaar, 1989).

A bidirectional crosstalk was never encountered for channelpair (ctx4->rRTN). Therefore, it is likely that the rRTN acts as a resonator, which (passively) picks up the SWD activity sent by the somatosensory cortex. Still, the activation of the rRTN is apparently relevant for SWD maintenance and a reduction of this activity, brought about by a termination of the cortical drive and an increase in coupling strength from the cRTN, (see below) is involved in the termination of SWD. Activation of the rRTN, via stimulation of the ATN, prolonged the duration of both stimulation induced AD as well as spontaneous SWD (Lüttjohann and van Luijtelaar, 2013).

The relevance of the RTN for SWD maintenance was already indicated in the late eighties and early nineties by lesion and pharmacological inhibition studies (Buzsaki et al., 1988; Avanzini et al., 1992, 1993). Also several changes have been reported in the RTN of absence epileptic rats, such as a specific GABA α3 subunit loss in WAG/Rij rats (Liu et al., 2007), an increase in a subtype high voltage Ca^2+^ channel subunit, again in WAG/Rij rats (Van De Bovenkamp-Janssen et al., 2004) and an increase in the T-type Ca^2+^ conductance in GAERS (Tsakiridou et al., 1995). The involvement of the rRTN in the control of the duration (termination) of the spontaneously occurring SWDs was demonstrated by us in freely moving rats. The above mentioned changes might explain the susceptibility of the rRTN to function as a resonator for SWD maintenance in these genetic epileptic rats.

#### The caudal reticular thalamic nucleus, a “gate keeper”

The network behavior of the cRTN can best be interpreted as representing a “gate-keeper” or “break” for SWD activity. During the pre-FCTS interval the cRTN decouples from the VPM (trigger input relay), cortical layer IV (the major sensory input layer of the somatosensory cortex) and the reverberator Po (Lüttjohann et al., 2013). The relevance of such a decoupling for SWD generation was described by Amor et al. (2009) and Le Van Quyen (2005). They detected a pre-ictal decrease in cortical long range synchronization in patients with SWD (juvenile absence epilepsy) (Amor et al., 2009). The authors assume that such a desynchronization might prepare a brain network toward a pro-epileptic state, since such a desynchronization might reflect a depression of synaptic inhibition and might provide an “idle” population of neurons that can easily be recruited by an epileptic focus. In line with this interpretation, the decoupling of the inhibitory cRTN might also bring the cortico-thalamo-cortical network into a pro-epileptic state, and this might be a prerequisite for SWD generation. As a consequence of a reduced inhibitory influence of the cRTN onto the Po, a population of “idle” Po neurons might be created, which then allows the epileptic focus to increase its communication to the important “reverberator,” the Po. Secondly, the short lasting reduction of “gate-keeping/gate closing” activity of the cRTN on the VPM input relay and cortical input layer IV might change the way in which an external stimulus (or electrical pulse provided Lüttjohann et al., 2013), might reach the hyperexcitable, focal, cortical area. It is known that during low levels of vigilance the increased inhibition of the cRTN onto thalamic relay cells, like the VPM, reduces/blocks the amount of externally relayed signal to the cortex as expressed in a reduced thalamic transfer ratio (Coenen, 1995). Furthermore, it is known that during a low level of vigilance, cortical cells react in a synchronous burst like fashion toward an incoming stimulus (Coenen, 1995). The short lasting decoupling of the cRTN might now allow an external stimulus to reach the epileptic focal zone and functions as an effective trigger for SWD initiation.

Our assumption, that a decoupling and its resulting preparation of the system into a pro-epileptic state is a prerequisite for SWD generation, might be supported by the fact that such a decoupling in patients with focal epilepsy could already be used to predict seizures with a sensitivity of up to 100% (Mormann et al., 2003).

While the pre-ictal decoupling of the cRTN might be regarded as the removal of a break for SWD activity, the function of the break, as attributed here to the cRTN, becomes especially visible during the “pre-LCTS” period. The process of SWD termination is initiated by a strong increase in directional coupling of the cRTN onto the rRTN. It is assumed that this represents an increased inhibition onto the rRTN and that this might, like the decreased cortical drive to this nucleus as discussed above, result in a decreased activity of the “rRTN resonator,” important for SWD maintenance. It is therefore that we propose that the cRTN breaks the resonating activity of the rRTN and therefore initiates SWD termination (Lüttjohann et al., 2014). Pharmacological *in vivo* and *in vitro* studies have suggested that intra-RTN communication is involved in the control of the duration of SWD (Sohal et al., 2003; Proulx et al., 2006). Again, our signal analytical data support the idea about the function of the cRTN and it is, to the best of our knowledge, the first time that this function is proposed based on data as obtained in a natural (not manipulated) context of spontaneously occurring and terminating SWD.

## Imaging studies in children with childhood absence epilepsy

Different imaging techniques (EEG, MEG, fMRI) have been used to investigate brain structures involved in the generation and maintenance of SWD, including the network dynamics, the possibility for the existence of a focal, epileptic onset zone and the existence of some sort of precursor activity. For the ease of comparison between species, this section focuses on human data acquired in children with Childhood Absence Epilepsy (CAE), while network studies analyzing SWD recordings from other epilepsy syndromes like Juvenile Absence Epilepsy, Myoclonic Absence epilepsy and Juvenile Myoclonic Epilepsy are not considered. For the sake of being complete, children with CAE show SWD with a frequency of 2.5–4 Hz, while in the genetic rodent models SWD have a frequency of 6–11 Hz. It is not clear to which extend this difference in frequency reflects differences in the mechanisms of SWD synchronization between species. Currently, the most excepted explanation for this species difference is a difference GABA-ergic conductance profiles within the cortico-thalamo-cortical system with humans possessing a GABA_B_ conduction dominance and rodents possessing a GABA_A_ conductiondominance (Destexhe, 1999). Alternatively, a simple difference in brain size has been proposed as a potential explanation (with longer axons and dendrites of CT and TC cells in humans as compared to rats and thus longer signal-conduction times) (Roberts and Robinson, 2008).

**Figure 7:**
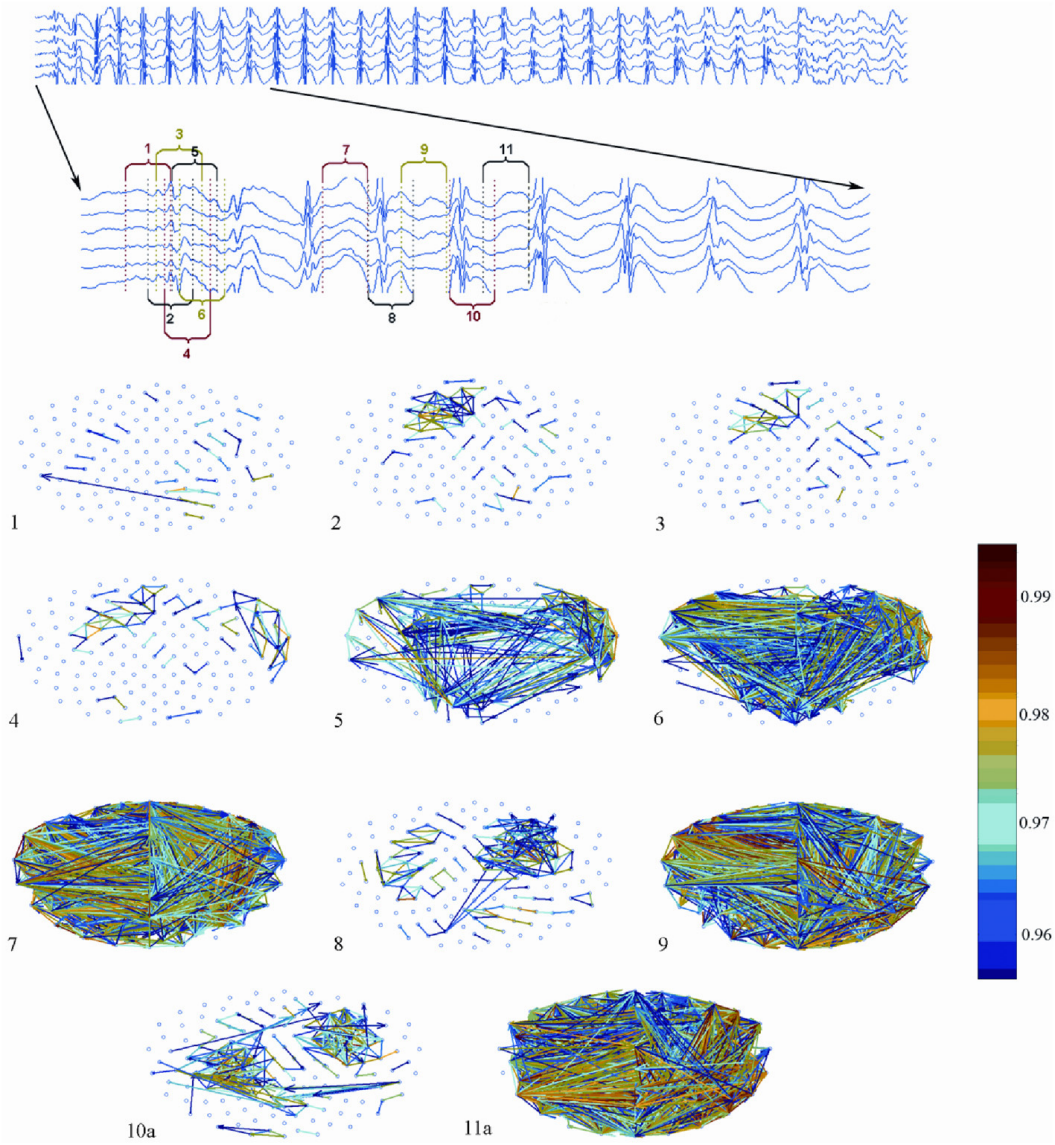
**Association strength between MEG sensors assessed across SWD onset with the aid of non-linear association analysis**. Note the local clusters accompanying the spike component of SWD and the broad generalization accompanying the wave components of SWD as well as the early, local cluster of connectivity preceeding the first generalized spike. Figure adapted from Westmijse et al. (2009).

Westmijse et al. (2009) used the same non-linear association analysis as used in the Meeren et al. (2002) rat study for the analysis of pre-SWD -> SWD transition periods of MEG recorded SWD from five, medicated, children. They discovered focal clusters of highly synchronized activity preceding SWD onset that were consistently located in frontal cortical regions while sources of the spikes from a train of SWDs were at the frontal lateral, central and medial parietal cortices, as confirmed by a beamforming source-localization technique. Next a repetitive alteration between 1 and 2 focal clusters of high associations, seen during each spike of SWDs and a broad generalization (high association strength between all MEG sensors) during the wave component of the SWD was found (Figure 7).

The local clusters seen during the spike component were considered to support the idea that also in CAE patients, SWD are not primary generalized oscillations. Instead, SWD have a local, cortical, onset zone and this is in line with the cortical focus theory. The repetitive nature of local clusters with high synchronization during the spike and broad generalization during the wave was interpreted as a cyclic interaction of propagating activity between cortex and thalamus (Westmijse et al., 2009; Ossenblok et al., 2013). Neither the non-linear association analysis nor the beamforming technique, applied in this study, was suited to detect activity of deep brain structures like the thalamus, so that the possibility of a leading thalamus, which would falsify the cortical focus theory for the patients, could not be excluded (Westmijse et al., 2009).

The involvement of the thalamus, as part of the SWD network, was demonstrated in combined EEG-fMRI studies by Moeller et al. (Moeller et al., 2008, 2010). Here, increases in thalamic bold activity was consistently found in 94% of SWD together with patient specific, localized cortical increases as well as decreased BOLD responses in parietal regions, precuneus and the caudate nucleus (default mode areas). Interestingly, the patient specific cortical activations occurred significantly earlier than the thalamic ones. In addition, patterns of BOLD activation were highly consistent between SWD within the same patient (Moeller et al., 2010).

The earlier cortical activation as compared to the thalamic one was also confirmed in a study by Bai et al. (2010), who performed simultaneous EEG/fMRI recordings in 9 children with CAE (88 analyzed SWD). Interestingly, in contrast to prior EEG/fMRI studies these early, cortical, seizure related increases, seen as local clusters mostly located within orbital/medial frontal cortex and medial/lateral parietal cortex, were already seen to occur up to 5 s prior to SWD onset. The Bai et al. study is the first that demonstrates the existence of SWD precursor activity in fMRI recordings and it supports the idea, that SWD are not sudden and unpredictable. The lack of sensitivity to detect precursor activity in other EEG/fMRI studies might be attributed to a difference in signal analysis. In contrast to others, Bai et al. (2010) did not employ standard Hemodynamic Response Function modeling, which possess several a priori assumptions about the data. Instead, they constructed maps of mean percentage change in fMRI signal on a voxel-by-voxel basis throughout the brain at different SWD time points.

The activity pattern of different thalamic nuclei, so far, was only investigated by a single EEG/fMRI study performed (Tyvaert et al., 2009). In contrast to the other studies discussed in this section, MAE and JME patients were included. Most fMRI studies only report activity changes in the thalamus as a single, unified entity; Tyvaert et al. compared the time course of BOLD changes in two thalamic nuclei, the anterior thalamic nuclei and the Centromedian-Parafasicular (CM-Pf) nuclear complex, during the generation of SWD. They reported a significantly earlier increase in BOLD response for the CM-Pf as compared to the anterior thalamic nucleus. Interestingly like the Po, seen to show earliest thalamic activity in our rat studies (Lüttjohann and van Luijtelaar, 2012), the CM-Pf is also a higher order nucleus, which receives its major input from the cortex. The first order anterior thalamic nucleus, like all first order thalamic nuclei in the rat studies, on the other hand, show later increases in SWD related activity (i.e., coupling with the cortex in the rat studies). As mentioned already in the section on the animal data, higher order nuclei are not only characterized by receiving their driving input from the cortex (and by that probably from a cortical epileptic focus), but also possess the characteristic, that they send back thalamo-cortical projections to multiple distributed cortical areas instead of to a single specific one (Sherman and Guillery, 2005). These characteristics, together with an early activation, probably make them suitable to be involved in the rapid generalization of the SWD (Kostopoulos, 2001; Polack et al., 2007; Lüttjohann and van Luijtelaar, 2012).

On the other hand, the above described fMRI results about differences in timing of BOLD activation of a brain structure (i.e., a local cortical area) does not imply that the cortex is “leading” the other structures. The inherently low time resolution of the BOLD signal makes it a less useful technique to determine rapid time sequences of the underlying electrical events, which occur at much faster timescales (Logothetis et al., 2001; Tenney et al., 2013).

A better temporal resolution can be achieved in EEG/MEG studies although with the trade-off of the spatial resolution. Moreover, it has been doubted whether it is possible to detected sources of activity within the thalamus with the aid of EEG/MEG source modeling techniques.

An elegant study by Moeller et al. (2012), on the other hand, nicely demonstrated that this is possible in the case of absence seizures. These authors performed EEG/fMRI recordings of 10 children with CAE and performed source analysis on the EEG data with the aid of the Dynamic Imaging of Coherent Sources (DICS) technique (Gross et al., 2001). Comparison between the by DICS detected sources of activity and the locations showing SWD related BOLD changes revealed a strong similarity, except for some differences in between patient variability. Of note, the strongest concordance between both methods was revealed for the thalamus. Detection of the neuronal sources by DICS allowed the authors to perform a directed connectivity analysis, a renormalized partial directed coherence analysis in source space. For the five main sources detected by DICS (medial prefrontal cortex, lateral prefrontal cortex, parietal cortex, lateral occipital cortex and medial thalamus) this analysis revealed that the medial prefrontal cortex guided the other cortical sites, however that the thalamic source influenced all cortical sources. While this at first glance appears to be contradictory to the cortical focus theory, it needs to be mentioned that Moeller and colleagues performed their source analysis and Partial Directed Coherence analysis on whole SWD epochs, and did not analyze temporal dynamics in directed connectivity. A prerequisite of a cortical focus, also in children with CAE, is that a local cortical source needs to guide other brain areas at the beginning of an SWD, while thereafter, as also described in the rat studies (Meeren et al., 2002), a constantly guiding cortex is not required.

Another MEG study using DICS was performed by Gupta et al. (2011). They combined it with a non-linear association analysis to assess connectivity between sensors and further processed their connectivity results in the framework of Graph theory. They assessed cluster coefficients (CC), an estimate for local connectivity, and the characteristic path length (CPL), defined as the mean of all shortest paths between all pairs of MEG sensors, which is seen as an indicator for global connectivity.

In contrast to Moeller, these authors took the time course of SWD generation into account and by this were able to detect early precursor activity in the delta frequency range in frontal cortical and occipital sources preceding the first spike of the SWD. A pre-SWD preparation state was also confirmed, showing an increase in clustering and a decrease in path length from the inter-ictal to the pre-ictal state. This confirms that, like in the rat models, SWD as seen in children with CAE, can no longer be regarded as sudden and unpredictable.

Last but not least we want to highlight a recent MEG study by Tenney et al. (2013). In their studies SWD recorded in 12 newly diagnosed, drug naïve, children with CAE (44 analyzed SWD) were analyzed with the aid of two different source analysis methods (SAM Beamforming and s'Loreta). In the SAM-Beamforming virtual sensors were placed throughout the thalamus and the cortical surface to investigate the possibility to detect independent thalamic and cortical activity during SWD and to determine the relative contribution of these sources. s'Loreta was used to compute statistical maps indicating MEG source locations during the time course of the SWD. For the latter an analysis window of 25 ms was shifted in steps of 25 ms along a pre-SWD->SWD transition period of 300 ms starting 50 ms prior to SWD onset, as well as along a “mid-ictal interval” approximately 3 s later, surrounding (50 ms pre and 250 ms post) the average time point of clinical symptom onset (Bai et al., 2010). As in the study by Moeller et al., also in the Tenney study beamforming detected thalamic activity, which was clearly distinct from cortical activity as indicated by a different morphology, timing and amplitude. Statistical group analysis of the time course of activity (as revealed by s'Loreta) revealed that in 70% of SWD local (source) activity in the frontal cortex and the thalamus was already seen prior (−50 to −25 ms) to SWD onset, which was followed by a source localization, almost completely restricted to the frontal cortex at SWD onset. Thereafter source localization was more widespread across several cortical areas. Statistical comparison between the ictal-onset and the mid-ictal period demonstrated significant differences for source localizations during the wave components of the SWD with wave components during the ictal onset showing a stronger frontal and thalamic localization profile and wave components during the mid-ictal period showing a more predominant parietal-temporal localization profile (Tenney et al., 2013).

Human and animal studies accumulate evidence that in both species SWD onset is preceded by SWD preparatory processes both locally and in changes in network activity. Both in humans and in rats, the detection of pre-ictal activity depends to some degree on the type of analysis applied. In addition, SWD and also other so-called generalized seizures have a focal onset and should no longer be considered as primarily generalized seizures (van Luijtelaar et al., 2014). Three characteristics of a focal type of epilepsy were present in the rat data. Data in humans still remain on a descriptive level, although the pre-ictal localized cortical activity is a strong indication in favor for the existence of a cortical epileptic focus, however a directed connectivity analysis along the time-course of a pre-SWD -> SWD transition period, is still missing. This analyses could investigate if the local cortical source “drives” other sources, located elsewhere, at the onset of SWD.

The most obvious difference between rat and human data is the location of the early local cortical activity. Whereas in the rat the epileptic focus is always located within the perioral somatosensory cortex (WAG/Rij) or the secondary somatosensory cortex (GAERS), the local cortical onset zone in children with CAE is more variable between subjects, might even slightly change in position during a seizure as indicated by Westmijse et al. (2009) and is predominantly but not exclusive located in the frontal—central/parietal areas. The less variability in the rats can easily be explained by the fact that both epileptic strains are fully inbred and the animals are homozygous. The location of the epileptic focus in the somatosensory cortex was linked to the fact that epileptic rats are albino rats, in which the sensory system of the vibrissae might be more sensitive and hyperexcitable to compensate for a deteriorated visual system (van Luijtelaar and Sitnikova, 2006).

Independent of the location of the cortical onset zone, Lüttjohann et al. (2013) proposed that the network mechanisms for SWD generation, as described in rats, might be rather similar for different species. A pre-SWD change in the inhibitory and excitatory balance in the connected brain network (decoupling of the RTN), enables a situation of increased communication between a susceptible zone and its anatomically connected thalamic counterparts (prerequisite 1). It enables that also a trigger-input can reach this susceptible zone (prerequisite 2) starting a localized epileptic discharge that is picked up by the cortex, and spread over the cortex and to the thalamus in order to become quickly generalized. Whether in the humans this thalamic counterpart is also a higher order thalamic nucleus (as the Po for the WAG/Rij rat) needs to be investigated but might be favored by the results of Tyvaert et al. (2009), described above. The importance of the different thalamic nuclei in transmitting a trigger pulse to the cortex, for being a resonator and reverberator and playing a role in maintenance in humans can be extracted from the consistent but differential activation patterns seen in the fMRI and some source modeling studies (Moeller et al., 2008, 2012; Tyvaert et al., 2009; Bai et al., 2010; Tenney et al., 2013).

## Epilog: SWD—more than a cortical focus

The advent of the cortical focus theory in 2002, stating that the deep somatosensory cortex of absence epileptic rats contains a local epileptic focus, which is the origin of SWD, while the thalamus, functions as a resonator for SWD maintenance, has led to a shift in the distribution of functional significance of cortex and thalamus. It changed from an almost equal relevance of cortex and thalamus in the cortico-reticular theory (i.e., attributing the thalamus with the function of the rhythm generator and the cortex with the function of a transformer (see Section Introduction) to a much stronger emphasis on the somatosensory cortex in the cortical focus theory. As a consequence, the attention of subsequently performed research in rodents shifted toward the somatosensory cortex.

While in many experiments within the last years (for review see e.g., van Luijtelaar and Sitnikova, 2006; van Luijtelaar et al., 2011b) as well as in experiments summarized in this review quite some evidence in favor of the deep somatosensory cortex as initiator of SWD was obtained (see also Section Different Functional Contributions of Network Structures for SWD Generation, Maintenance and Termination), the present review also summarizes results which demonstrate that the thalamus is more than just a “passive” resonator for SWD maintenance. Early pre-ictal changes in cortico-thalamic as well as intra-thalamic coupling prepare the system toward a proepileptic state and can be held responsible for whether the cortical focal area gets the chance to entrain a larger part of the cortex in SWD activity and determine whether a “full-blown” cortico-thalamo-cortical SWD can arise.

Avoli (2012), giving a short historical overview on the theories of SWD generation, noticed that the significance which is given to the cortex or thalamus in SWD generation has proceeded in cycles from thalamus (centrencephalic theory) to cortex (cortical theory), to thalamus and cortex (cortico-reticular theory, and back to cortex (cortical-focus theory), and predicted that the next epoch will again be a thalamic one. The description of e.g., the Posterior thalamic nucleus, as an important “reverberator,” crucially relevant for SWD generation (see Section Different Functional Contributions of Network Structures for SWD Generation, Maintenance and Termination) might be in line with his prediction. However, the authors of this review want to disagree or better warn for such a development. Local changes have an effect on a complete network and certainly in a dense easily reverberating network called the cortico-thalamo-cortical circuit (van Luijtelaar et al., 2014). Therefore, the most appropriate way to learn about SWD generation, generalization, maintenance, and termination are studies investigating network interactions, irrespective of the sites of origin.

### Conflict of interest statement

The authors declare that the research was conducted in the absence of any commercial or financial relationships that could be construed as a potential conflict of interest.

## References

[B1] Abbasova K. R. Chepurnov S. A. Chepurnova N. E. van Luijtelaar G. (2010). The role of perioral afferentation in the occurrence of spike-wave discharges in the WAG/Rij model of absence epilepsy. Brain Res. 1366, 257–262. 10.1016/j.brainres.2010.10.007 20934415

[B2] Aker R. G. Ozyurt H. B. Yananli H. R. Cakmak Y. O. Ozkaynakci A. E. Sehirli U.. (2006). GABA(A) receptor mediated transmission in the thalamic reticular nucleus of rats with genetic absence epilepsy shows regional differences: functional implications. Brain Res. 1111, 213–221. 10.1016/j.brainres.2006.06.118 16919245

[B3] Akman O. Demiralp T. Ates N. Onat F. Y. (2010). Electroencephalographic differences between WAG/Rij and GAERS rat models of absence epilepsy. Epilepsy Res. 89, 185–193. 10.1016/j.eplepsyres.2009.12.005 20092980

[B4] Amor F. Baillet S. Navarro V. Adam C. Martinerie J. Quyen Mle V. (2009). Cortical local and long-range synchronization interplay in human absence seizure initiation. Neuroimage 45, 950–962. 10.1016/j.neuroimage.2008.12.011 19150654

[B5] Avanzini G. De Curtis M. Marescaux C. Panzica F. Spreafico R. Vergnes M. (1992). Role of the thalamic reticular nucleus in the generation of rhythmic thalamo-cortical activities subserving spike and waves. J. Neural Transm. Suppl. 35, 85–95. 1512596 10.1007/978-3-7091-9206-1_6

[B6] Avanzini G. Vergnes M. Spreafico R. Marescaux C. (1993). Calcium-dependent regulation of genetically determined spike and waves by the reticular thalamic nucleus of rats. Epilepsia 34, 1–7. 10.1111/j.1528-1157.1993.tb02369.x 8422841

[B7] Avoli M. (2012). A brief history on the oscillating roles of thalamus and cortex in absence seizures. Epilepsia 53, 779–789. 10.1111/j.1528-1167.2012.03421.x 22360294 PMC4878899

[B8] Avoli M. Gloor P. (1981). The effects of transient functional depression of the thalamus on spindles and on bilateral synchronous epileptic discharges of feline generalized penicillin epilepsy. Epilepsia 22, 443–452. 10.1111/j.1528-1157.1981.tb06155.x 7262050

[B9] Avoli M. Rogawski M. A. Avanzini G. (2001). Generalized epileptic disorders: an update. Epilepsia 42, 445–457. 10.1046/j.1528-1157.2001.39800.x 11440339

[B10] Bai X. Vestal M. Berman R. Negishi M. Spann M. Vega C.. (2010). Dynamic time course of typical childhood absence seizures: EEG, behavior, and functional magnetic resonance imaging. J. Neurosci. 30, 5884–5893. 10.1523/JNEUROSCI.5101-09.2010 20427649 PMC2946206

[B11] Blumenfeld H. Klein J. P. Schridde U. Vestal M. Rice T. Khera D. S.. (2008). Early treatment suppresses the development of spike-wave epilepsy in a rat model. Epilepsia 49, 400–409. 10.1111/j.1528-1167.2007.01458.x 18070091 PMC3143182

[B12] Bosnyakova D. Gabova A. Kuznetsova G. Obukhov Y. Midzyanovskaya I. Salonin D.. (2006). Time-frequency analysis of spike-wave discharges using a modified wavelet transform. J. Neurosci. Methods 154, 80–88. 10.1016/j.jneumeth.2005.12.006 16434106

[B13] Bourassa J. Pinault D. Deschenes M. (1995). Corticothalamic projections from the cortical barrel field to the somatosensory thalamus in rats: a single-fibre study using biocytin as an anterograde tracer. Eur. J. Neurosci. 7, 19–30. 10.1111/j.1460-9568.1995.tb01016.x 7711933

[B14] Buzsaki G. Bickford R. G. Ponomareff G. Thal L. J. Mandel R. Gage F. H. (1988). Nucleus basalis and thalamic control of neocortical activity in the freely moving rat. J. Neurosci. 8, 4007–4026. 3183710 10.1523/JNEUROSCI.08-11-04007.1988 PMC6569493

[B15] Cavdar S. Hacioglu H. Dogukan S. Y. Onat F. (2012). Do the quantitative relationships of synaptic junctions and terminals in the thalamus of genetic absence epilepsy rats from Strasbourg (GAERS) differ from those in normal control Wistar rats. Neurol. Sci. 33, 251–259. 10.1007/s10072-011-0666-5 21720899

[B16] Coenen A. M. (1995). Neuronal activities underlying the electroencephalogram and evoked potentials of sleeping and waking: implications for information processing. Neurosci. Biobehav. Rev. 19, 447–463. 10.1016/0149-7634(95)00010-C 7566746

[B17] Coenen A. M. van Luijtelaar E. L. (1989). Effects of diazepam and two beta-carbolines on epileptic activity and on EEG and behavior in rats with absence seizures. Pharmacol. Biochem. Behav. 32, 27–35. 10.1016/0091-3057(89)90206-2 2734337

[B18] Coenen A. M. van Luijtelaar E. L. (2003). Genetic animal models for absence epilepsy: a review of the WAG/Rij strain of rats. Behav. Genet. 33, 635–655. 10.1023/A:1026179013847 14574120

[B19] Connors B. W. Gutnick M. J. (1990). Intrinsic firing patterns of diverse neocortical neurons. Trends Neurosci. 13, 99–104. 10.1016/0166-2236(90)90185-D 1691879

[B20] D'Amore V. Santolini I. Van Rijn C. M. Biagioni F. Molinaro G. Prete A.. (2013). Potentiation of mGlu5 receptors with the novel enhancer, VU0360172, reduces spontaneous absence seizures in WAG/Rij rats. Neuropharmacology 66, 330–338. 10.1016/j.neuropharm.2012.05.044 22705340 PMC3787880

[B21] D'antuono M. Inaba Y. Biagini G. D'arcangelo G. Tancredi V. Avoli M. (2006). Synaptic hyperexcitability of deep layer neocortical cells in a genetic model of absence seizures. Genes Brain Behav. 5, 73–84. 10.1111/j.1601-183X.2005.00146.x 16436191

[B22] Danober L. Deransart C. Depaulis A. Vergnes M. Marescaux C. (1998). Pathophysiological mechanisms of genetic absence epilepsy in the rat. Prog. Neurobiol. 55, 27–57. 10.1016/S0301-0082(97)00091-9 9602499

[B23] David O. Guillemain I. Saillet S. Reyt S. Deransart C. Segebarth C.. (2008). Identifying neural drivers with functional MRI: an electrophysiological validation. PLoS Biol. 6:e315. 10.1371/journal.pbio.0060315 19108604 PMC2605917

[B24] Depaulis A. van Luijtelaar G. (2006). Genetic models of Absence epilepsy, in Models of Seizures and Epilepsy, eds Pitkanen A. Schwartzkroin P. A. Moshe S. L. (San Diego, CA: Elsevier Academic Press), 233–248.

[B25] Deschênes M. Bourassa J. Pinault D. (1994). Corticothalamic projections from layer V cells in rat are collaterals of long-range corticofugal axons. Brain Res. 664, 215–219. 10.1016/0006-8993(94)91974-7 7895031

[B26] Deschênes M. Veinante P. Zhang Z. W. (1998). The organization of corticothalamic projections: reciprocity versus parity. Brain Res. Brain Res. Rev. 28, 286–308. 10.1016/S0165-0173(98)00017-4 9858751

[B27] Destexhe A. (1999). Can GABAA conductances explain the fast oscillation frequency of absence seizures in rodents? Eur. J. Neurosci. 11, 2175–2181. 10.1046/j.1460-9568.1999.00660.x 10336687

[B28] Dhamala M. Rangarajan G. Ding M. (2008). Analyzing information flow in brain networks with nonparametric Granger causality. Neuroimage 41, 354–362. 10.1016/j.neuroimage.2008.02.020 18394927 PMC2685256

[B29] Drinkenburg W. H. Coenen A. M. Vossen J. M. van Luijtelaar E. L. (1991). Spike-wave discharges and sleep-wake states in rats with absence epilepsy. Epilepsy Res. 9, 218–224. 10.1016/0920-1211(91)90055-K 1743184

[B30] Gloor P. (1968). Generalized cortico-reticular epilepsies. Some considerations on the pathophysiology of generalized bilaterally synchronous spike and wave discharge. Epilepsia 9, 249–263. 10.1111/j.1528-1157.1968.tb04624.x 4975028

[B31] Gloor P. Avoli M. Kostopoulos G. (1990). Thalamo-cortical relationships in generalized epilepsy with bilaterally synchronous spike-wave discharge, in Generalized Epilepsy: Neurobiological Approaches, eds Avoli M. Gloor P. Naquet R. Kostopoulos G. (Boston, MA: BirkhaÈuser Boston Inc), 190–212.

[B32] Golshani P. Jones E. G. (1999). Synchronized paroxysmal activity in the developing thalamocortical network mediated by corticothalamic projections and “silent” synapses. J. Neurosci. 19, 2865–2875. 10191304 10.1523/JNEUROSCI.19-08-02865.1999 PMC6782276

[B33] Gonzalo-Ruiz A. Lieberman A. R. (1995). Topographic organization of projections from the thalamic reticular nucleus to the anterior thalamic nuclei in the rat. Brain Res. Bull. 37, 17–35. 10.1016/0361-9230(94)00252-5 7606476

[B34] Goodfellow M. Taylor P. N. Wang Y. Garry D. J. Baier G. (2012). Modelling the role of tissue heterogeneity in epileptic rhythms. Eur. J. Neurosci. 36, 2178–2187. 10.1111/j.1460-9568.2012.08093.x 22805063

[B35] Gross J. Kujala J. Hamalainen M. Timmermann L. Schnitzler A. Salmelin R. (2001). Dynamic imaging of coherent sources: studying neural interactions in the human brain. Proc. Natl. Acad. Sci. U.S.A. 98, 694–699. 10.1073/pnas.98.2.694 11209067 PMC14650

[B36] Gupta D. Ossenblok P. van Luijtelaar G. (2011). Space-time network connectivity and cortical activations preceding spike wave discharges in human absence epilepsy: a MEG study. Med. Biol. Eng. Comput. 49, 555–565. 10.1007/s11517-011-0778-3 21533620

[B37] Gurbanova A. A. Aker R. Berkman K. Onat F. Y. van Rijn C. M. van Luijtelaar G. (2006). Effect of systemic and intracortical administration of phenytoin in two genetic models of absence epilepsy. Br. J. Pharmacol. 148, 1076–1082. 10.1038/sj.bjp.0706791 16865096 PMC1752009

[B38] Huguenard J. R. McCormick D. A. (2007). Thalamic synchrony and dynamic regulation of global forebrain oscillations. Trends Neurosci. 30, 350–356. 10.1016/j.tins.2007.05.007 17544519

[B39] ILAE. (1981). Proposal for revised clinical and electroencephalographic classification of epileptic seizures. From the commission on classification and terminology of the international league against epilepsy. Epilepsia 22, 489–501. 6790275 10.1111/j.1528-1157.1981.tb06159.x

[B40] Inaba Y. D'antuono M. Bertazzoni G. Biagini G. Avoli M. (2009). Diminished presynaptic GABA(B) receptor function in the neocortex of a genetic model of absence epilepsy. Neurosignals 17, 121–131. 10.1159/000197864 19176980 PMC4878904

[B41] Jasper H. H. Fortuyn J. D. (1947). Experimental studies on the functional anatomy of petit mal epilepsy. Res. Publ. Assoc. Res. Nerv. Ment. Dis. 26, 26272–26298.

[B42] Jones E. G. (2009). Synchrony in the interconnected circuitry of the thalamus and cerebral cortex. Ann. N.Y. Acad. Sci. 1157, 10–23. 10.1111/j.1749-6632.2009.04534.x 19351352

[B43] Killackey H. P. Sherman S. M. (2003). Corticothalamic projections from the rat primary somatosensory cortex. J. Neurosci. 23, 7381–7384. 12917373 10.1523/JNEUROSCI.23-19-07381.2003 PMC6740432

[B44] Klein J. P. Khera D. S. Nersesyan H. Kimchi E. Y. Waxman S. G. Blumenfeld H. (2004). Dysregulation of sodium channel expression in cortical neurons in a rodent model of absence epilepsy. Brain Res. 1000, 102–109. 10.1016/j.brainres.2003.11.051 15053958

[B45] Kole M. H. Brauer A. U. Stuart G. J. (2007). Inherited cortical HCN1 channel loss amplifies dendritic calcium electrogenesis and burst firing in a rat absence epilepsy model. J. Physiol. 578, 507–525. 10.1113/jphysiol.2006.122028 17095562 PMC2075144

[B46] Kostopoulos G. K. (2001). Involvement of the thalamocortical system in epileptic loss of consciousness. Epilepsia 42(Suppl. 3), 13–19. 10.1046/j.1528-1157.2001.042suppl.3013.x 11520316

[B49] Lehnertz K. Widman G. Andrzejak R. Arnhold J. Elger C. E. (1999). Is it possible to anticipate seizure onset by non-linear analysis of intracerebral EEG in human partial epilepsies? Rev. Neurol. (Paris) 155, 454–456. 10472658

[B50] Leresche N. Lambert R. C. Errington A. C. Crunelli V. (2012). From sleep spindles of natural sleep to spike and wave discharges of typical absence seizures: is the hypothesis still valid? Pflugers Arch. 463, 201–212. 10.1007/s00424-011-1009-3 21861061 PMC3256322

[B47] Le Van Quyen M. (2005). Anticipating epileptic seizures: from mathematics to clinical applications. C. R. Biol. 328, 187–198. 10.1016/j.crvi.2004.10.014 15771005

[B48] Le Van Quyen M. Martinerie J. Baulac M. Varela F. (1999). Anticipating epileptic seizures in real time by a non-linear analysis of similarity between EEG recordings. Neuroreport 10, 2149–2155. 10.1097/00001756-199907130-00028 10424690

[B51] Litt B. Echauz J. (2002). Prediction of epileptic seizures. Lancet Neurol. 1, 22–30 10.1016/S1474-4422(02)00003-0 12849542

[B52] Liu X. B. Coble J. van Luijtelaar G. Jones E. G. (2007). Reticular nucleus-specific changes in alpha3 subunit protein at GABA synapses in genetically epilepsy-prone rats. Proc. Natl. Acad. Sci. U.S.A. 104, 12512–12517. 10.1073/pnas.0705320104 17630284 PMC1916487

[B53] Logothetis N. K. Pauls J. Augath M. Trinath T. Oeltermann A. (2001). Neurophysiological investigation of the basis of the fMRI signal. Nature 412, 150–157. 10.1038/35084005 11449264

[B54] Lopes Da Silva F. Blanes W. Kalitzin S. N. Parra J. Suffczynski P. Velis D. N. (2003a). Epilepsies as dynamical diseases of brain systems: basic models of the transition between normal and epileptic activity. Epilepsia 44(Suppl. 12), 72–83. 10.1111/j.0013-9580.2003.12005.x 14641563

[B55] Lopes Da Silva F. Pijn J. P. Boeijinga P. (1989). Interdependence of EEG signals: linear vs. nonlinear associations and the significance of time delays and phase shifts. Brain Topogr. 2, 9–18. 10.1007/BF01128839 2641479

[B56] Lopes Da Silva F. H. Blanes W. Kalitzin S. N. Parra J. Suffczynski P. Velis D. N. (2003b). Dynamical diseases of brain systems: different routes to epileptic seizures. IEEE Trans. Biomed. Eng. 50, 540–548. 10.1109/TBME.2003.810703 12769430

[B57] Lu S. M. Lin R. C. (1993). Thalamic afferents of the rat barrel cortex: a light- and electron-microscopic study using Phaseolus vulgaris leucoagglutinin as an anterograde tracer. Somatosens. Mot. Res. 10, 1–16. 10.3109/08990229309028819 8484292

[B58] Luhmann H. J. Mittmann T. van Luijtelaar G. Heinemann U. (1995). Impairment of intracortical GABAergic inhibition in a rat model of absence epilepsy. Epilepsy Res. 22, 43–51. 10.1016/0920-1211(95)00032-6 8565966

[B59] Lüttjohann A. Schoffelen J. M. van Luijtelaar G. (2013). Peri-ictal network dynamics of spike-wave discharges: phase and spectral characteristics. Exp. Neurol. 239, 235–247. 10.1016/j.expneurol.2012.10.021 23124095

[B60] Lüttjohann A. Schoffelen J. M. van Luijtelaar G. (2014). Termination of ongoing spike-wave discharges investigated by cortico-thalamic network analyses. Neurobiol. Dis. 70, 127–137. 10.1016/j.nbd.2014.06.007 24953875

[B61] Lüttjohann A. van Luijtelaar G. (2012). The dynamics of cortico-thalamo-cortical interactions at the transition from pre-ictal to ictal LFPs in absence epilepsy. Neurobiol. Dis. 47, 49–60. 10.1016/j.nbd.2012.03.023 22465080

[B62] Lüttjohann A. van Luijtelaar G. (2013). Thalamic stimulation in absence epilepsy. Epilepsy Res. 106, 136–145. 10.1016/j.eplepsyres.2013.03.009 23602552

[B63] Lüttjohann A. Zhang S. De Peijper R. van Luijtelaar G. (2011). Electrical stimulation of the epileptic focus in absence epileptic WAG/Rij rats: assessment of local and network excitability. Neuroscience 188, 125–134. 10.1016/j.neuroscience.2011.04.038 21569824

[B64] Marescaux C. Vergnes M. Depaulis A. (1992). Genetic absence epilepsy in rats from Strasbourg–a review. J. Neural Transm. Suppl. 35, 37–69. 1512594 10.1007/978-3-7091-9206-1_4

[B65] Maris E. Bouwman B. M. Suffczynski P. Van Rijn C. M. (2006). Starting and stopping mechanisms of absence epileptic seizures are revealed by hazard functions. J. Neurosci. Methods 152, 107–115. 10.1016/j.jneumeth.2005.08.016 16188323

[B66] Martinerie J. Adam C. Le Van Quyen M. Baulac M. Clemenceau S. Renault B.. (1998). Epileptic seizures can be anticipated by non-linear analysis. Nat. Med. 4, 1173–1176. 10.1038/2667 9771751

[B67] McCormick D. A. Contreras D. (2001). On the cellular and network bases of epileptic seizures. Annu. Rev. Physiol. 63, 815–846. 10.1146/annurev.physiol.63.1.815 11181977

[B68] Meeren H. van Luijtelaar G. Lopes Da Silva F. Coenen A. (2005). Evolving concepts on the pathophysiology of absence seizures: the cortical focus theory. Arch. Neurol. 62, 371–376. 10.1001/archneur.62.3.371 15767501

[B69] Meeren H. K. Pijn J. P. van Luijtelaar E. L. Coenen A. M. Lopes Da Silva F. H. (2002). Cortical focus drives widespread corticothalamic networks during spontaneous absence seizures in rats. J. Neurosci. 22, 1480–1495. 11850474 10.1523/JNEUROSCI.22-04-01480.2002 PMC6757554

[B70] Meeren H. K. Veening J. G. Moderscheim T. A. Coenen A. M. van Luijtelaar G. (2009). Thalamic lesions in a genetic rat model of absence epilepsy: dissociation between spike-wave discharges and sleep spindles. Exp. Neurol. 217, 25–37. 10.1016/j.expneurol.2009.01.009 19416679

[B71] Merlo D. Mollinari C. Inaba Y. Cardinale A. Rinaldi A. M. D'antuono M.. (2007). Reduced GABAB receptor subunit expression and paired-pulse depression in a genetic model of absence seizures. Neurobiol. Dis. 25, 631–641. 10.1016/j.nbd.2006.11.005 17207629

[B72] Moeller F. Levan P. Muhle H. Stephani U. Dubeau F. Siniatchkin M.. (2010). Absence seizures: individual patterns revealed by EEG-fMRI. Epilepsia 51, 2000–2010. 10.1111/j.1528-1167.2010.02698.x 20726875 PMC3769289

[B73] Moeller F. Muthuraman M. Stephani U. Deuschl G. Raethjen J. Siniatchkin M. (2012). Representation and propagation of epileptic activity in absences and generalized photoparoxysmal responses. Hum. Brain Mapp. 34, 1896–1909. 10.1002/hbm.22026 22431268 PMC6870437

[B74] Moeller F. Siebner H. R. Wolff S. Muhle H. Granert O. Jansen O.. (2008). Simultaneous EEG-fMRI in drug-naive children with newly diagnosed absence epilepsy. Epilepsia 49, 1510–1519. 10.1111/j.1528-1167.2008.01626.x 18435752

[B75] Mormann F. Andrzejak R. G. Kreuz T. Rieke C. David P. Elger C. E.. (2003). Automated detection of a preseizure state based on a decrease in synchronization in intracranial electroencephalogram recordings from epilepsy patients. Phys. Rev. E Stat. Nonlin. Soft Matter Phys. 67, 021912. 10.1103/PhysRevE.67.021912 12636720

[B76] Ngomba R. T. Biagioni F. Casciato S. Willems-Van Bree E. Battaglia G. Bruno V.. (2005). The preferential mGlu2/3 receptor antagonist, LY341495, reduces the frequency of spike-wave discharges in the WAG/Rij rat model of absence epilepsy. Neuropharmacology 49(Suppl. 1), 89–103. 10.1016/j.neuropharm.2005.05.019 16043198

[B77] Nicolelis M. A. L. Baccala L. A. Lin R. C. S. Chapin J. K. (1995). Sensorimotor encoding by synchronious neural ensemble activity at multiple level of the somatosensory system Science 268, 1353–1358. 10.1126/science.7761855 7761855

[B78] Oda S. Kishi K. Yang J. Chen S. Yokofujita J. Igarashi H.. (2004). Thalamocortical projection from the ventral posteromedial nucleus sends its collaterals to layer I of the primary somatosensory cortex in rat. Neurosci. Lett. 367, 394–398. 10.1016/j.neulet.2004.06.042 15337273

[B79] Ossenblok P. Houdt P. V. Lüttjohann A. van Luijtelaar G (2013). Network analysis of generalized epileptic discharges, in Preceedings of the 5th International Workshop on Seizure Prediction, eds Tetzlaff R. Lehnertz K. (Dresden).

[B80] Pijn J. P. Vijn P. C. Lopes Da Silva F. H. Van Ende Boas W. Blanes W. (1990). Localization of epileptogenic foci using a new signal analytical approach. Neurophysiol. Clin. 20, 1–11. 10.1016/S0987-7053(05)80165-0 2348808

[B81] Pinault D. (2004). The thalamic reticular nucleus: structure, function and concept. Brain Res. Brain Res. Rev. 46, 1–31. 10.1016/j.brainresrev.2004.04.008 15297152

[B82] Pinault D. O'Brien T. J. (2005). Cellular and network mechanisms of genetically-determined absence seizures. Thalamus Relat. Syst. 3, 181–203. 10.1017/S1472928807000209 21909233 PMC3168114

[B83] Pinault D. Slezia A. Acsady L. (2006). Corticothalamic 5-9 Hz oscillations are more pro-epileptogenic than sleep spindles in rats. J. Physiol. (Lond.) 574, 209–227. 10.1113/jphysiol.2006.108498 16627566 PMC1817782

[B84] Pinault D. Vergnes M. Marescaux C. (2001). Medium-voltage 5-9-Hz oscillations give rise to spike-and-wave discharges in a genetic model of absence epilepsy: *in vivo* dual extracellular recording of thalamic relay and reticular neurons. Neuroscience 105, 181–201. 10.1016/S0306-4522(01)00182-8 11483311

[B85] Polack P. O. Guillemain I. Hu E. Deransart C. Depaulis A. Charpier S. (2007). Deep layer somatosensory cortical neurons initiate spike-and-wave discharges in a genetic model of absence seizures. J. Neurosci. 27, 6590–6599. 10.1523/JNEUROSCI.0753-07.2007 17567820 PMC6672429

[B86] Polack P. O. Mahon S. Chavez M. Charpier S. (2009). Inactivation of the somatosensory cortex prevents paroxysmal oscillations in cortical and related thalamic neurons in a genetic model of absence epilepsy. Cereb. Cortex 19, 2078–2091. 10.1093/cercor/bhn237 19276326

[B87] Proulx E. Leshchenko Y. Kokarovtseva L. Khokhotva V. El-Beheiry M. Snead O. C. III. (2006). Functional contribution of specific brain areas to absence seizures: role of thalamic gap-junctional coupling. Eur. J. Neurosci. 23, 489–496. 10.1111/j.1460-9568.2005.04558.x 16420455

[B88] Pumain R. Louvel J. Gastard M. Kurcewicz I. Vergnes M. (1992). Responses to N-methyl-D-aspartate are enhanced in rats with petit mal-like seizures. J. Neural Transm. Suppl. 35, 97–108. 1512597 10.1007/978-3-7091-9206-1_7

[B89] Ramcharan E. J. Gnadt J. W. Sherman S. M. (2005). Higher-order thalamic relays burst more than first-order relays. Proc. Natl. Acad. Sci. U.S.A. 102, 12236–12241. 10.1073/pnas.0502843102 16099832 PMC1189315

[B90] Richards D. A. Manning J. P. Barnes D. Rombola L. Bowery N. G. Caccia S.. (2003). Targeting thalamic nuclei is not sufficient for the full anti-absence action of ethosuximide in a rat model of absence epilepsy. Epilepsy Res. 54, 97–107. 10.1016/S0920-1211(03)00060-3 12837561

[B91] Roberts J. A. Robinson P. A. (2008). Modeling absence seizure dynamics: implications for basic mechanisms and measurement of thalamocortical and corticothalamic latencies. J. Theor. Biol. 253, 189–201. 10.1016/j.jtbi.2008.03.005 18407293

[B92] Schridde U. Strauss U. Brauer A. U. van Luijtelaar G. (2006). Environmental manipulations early in development alter seizure activity, Ih and HCN1 protein expression later in life. Eur. J. Neurosci. 23, 3346–3358. 10.1111/j.1460-9568.2006.04865.x 16820024

[B93] Seidenbecher T. Staak R. Pape H. C. (1998). Relations between cortical and thalamic cellular activities during absence seizures in rats. Eur. J. Neurosci. 10, 1103–1112. 10.1046/j.1460-9568.1998.00123.x 9753178

[B94] Sherman S. M. Guillery R. W. (1998). On the actions that one nerve cell can have on another: distinguishing “drivers” from “modulators.” Proc. Natl. Acad. Sci. U.S.A. 95, 7121–7126. 10.1073/pnas.95.12.7121 9618549 PMC22761

[B95] Sherman S. M. Guillery R. W. (2005). Exploring the Thalamus and Its Role in Cortical Function. Cambridge: The MIT Press.

[B96] Sitnikova E. Dikanev T. Smirnov D. Bezruchko B. van Luijtelaar G. (2008). Granger causality: cortico-thalamic interdependencies during absence seizures in WAG/Rij rats. J. Neurosci. Methods 170, 245–254. 10.1016/j.jneumeth.2008.01.017 18313761

[B97] Sitnikova E. van Luijtelaar G. (2004). Cortical control of generalized absence seizures: effect of lidocaine applied to the somatosensory cortex in WAG/Rij rats. Brain Res. 1012, 127–137. 10.1016/j.brainres.2004.03.041 15158169

[B98] Sitnikova E. van Luijtelaar G. (2009). Electroencephalographic precursors of spike-wave discharges in a genetic rat model of absence epilepsy: power spectrum and coherence EEG analyses. Epilepsy Res. 84, 159–171. 10.1016/j.eplepsyres.2009.01.016 19269137

[B99] Sohal V. S. Keist R. Rudolph U. Huguenard J. R. (2003). Dynamic GABA(A) receptor subtype-specific modulation of the synchrony and duration of thalamic oscillations. J. Neurosci. 23, 3649–3657. 12736336 10.1523/JNEUROSCI.23-09-03649.2003 PMC6742195

[B100] Stefan H. Lopes Da Silva F. H. (2013). Epileptic neuronal networks: methods of identification and clinical relevance. Front. Neurol. 4:8. 10.3389/fneur.2013.00008 23532203 PMC3607195

[B101] Steriade M. (2003). Neuronal Substrates of Sleep and Epilepsy. Cambridge: Cambridge University Press.

[B102] Strauss U. Kole M. H. Bräuer A. U. Pahnke J. Bajorat R. Rolfs A.. (2004). An impaired neocortical Ih is associated with enhanced excitability and absence epilepsy. Eur. J. Neurosci. 19, 3048–3058. 10.1111/j.0953-816X.2004.03392.x 15182313

[B103] Sysoeva M. V. Sitnikova E. Sysoev I. V. Bezruchko B. P. van Luijtelaar G. (2014). Application of adaptive nonlinear Granger causality: disclosing network changes before and after absence seizure onset in a genetic rat model. J. Neurosci. Methods 226, 33–41. 10.1016/j.jneumeth.2014.01.028 24486875

[B104] Tenney J. R. Duong T. Q. King J. A. Ferris C. F. (2004). FMRI of brain activation in a genetic rat model of absence seizures. Epilepsia 45, 576–582. 10.1111/j.0013-9580.2004.39303.x 15144421 PMC2949946

[B105] Tenney J. R. Fujiwara H. Horn P. S. Jacobson S. E. Glauser T. A. Rose D. F. (2013). Focal corticothalamic sources during generalized absence seizures: a MEG study. Epilepsy Res. 106, 113–122. 10.1016/j.eplepsyres.2013.05.006 23764296

[B106] Thomson A. M. Bannister A. P. (2003). Interlaminar connections in the neocortex. Cereb. Cortex 13, 5–14. 10.1093/cercor/13.1.5 12466210

[B107] Timofeev I. Bazhenov M. Sejnowski T. J. Steriade M. (2001). Contribution of intrinsic and synaptic factors in the desynchronization of thalamic oscillatory activity. Thalamus Relat. Syst. 1, 53–69 10.1017/S1472928801000048

[B108] Tsakiridou E. Bertollini L. De Curtis M. Avanzini G. Pape H. C. (1995). Selective increase in T-type calcium conductance of reticular thalamic neurons in a rat model of absence epilepsy. J. Neurosci. 15, 3110–3117. 7722649 10.1523/JNEUROSCI.15-04-03110.1995 PMC6577780

[B109] Tyvaert L. Chassagnon S. Sadikot A. Levan P. Dubeau F. Gotman J. (2009). Thalamic nuclei activity in idiopathic generalized epilepsy: an EEG-fMRI study. Neurology 73, 2018–2022. 10.1212/WNL.0b013e3181c55d02 19996076

[B110] Van De Bovenkamp-Janssen M. C. Scheenen W. J. Kuijpers-Kwant F. J. Kozicz T. Veening J. G. van Luijtelaar E. L.. (2004). Differential expression of high voltage-activated Ca2+ channel types in the rostral reticular thalamic nucleus of the absence epileptic WAG/Rij rat. J. Neurobiol. 58, 467–478. 10.1002/neu.10291 14978724

[B114a] van de Bovenkamp-Janssen M. C. Van Der Kloet J. C. Van Luijtelaar G. Roubos E. W. (2006). NMDA-NR1 and AMPA-GluR4 receptor subunit immunoreactivities in the absence epileptic WAG/Rij rat. Epilepsy Res. 69, 119–128. 10.1016/j.eplepsyres.2006.01.003 16487682

[B111] van Luijtelaar G. Behr C. Avoli M. (2014). Is there such a thing as ‘generalized’ epilepsy?, in Issues in Clinical Epileptology: A View From the Bench, eds Scharfman H. E. Buckmaster P. (Dordrecht: Springer).

[B112] van Luijtelaar G. Hramov A. Sitnikova E. Koronovskii A. (2011a). Spike-wave discharges in WAG/Rij rats are preceded by delta and theta precursor activity in cortex and thalamus. Clin. Neurophysiol. 122, 687–695. 10.1016/j.clinph.2010.10.038 21093357

[B113] van Luijtelaar G. Mishra A. M. Edelbroek P. Coman D. Frankenmolen N. Schaapsmeerders P.. (2013). Anti-epileptogenesis: electrophysiology, diffusion tensor imaging and behavior in a genetic absence model. Neurobiol. Dis. 60, 126–138. 10.1016/j.nbd.2013.08.013 23978468 PMC3952020

[B114] van Luijtelaar G. Sitnikova E. (2006). Global and focal aspects of absence epilepsy: the contribution of genetic models. Neurosci. Biobehav. Rev. 30, 983–1003. 10.1016/j.neubiorev.2006.03.002 16725200

[B115] van Luijtelaar G. Sitnikova E. Lüttjohann A. (2011b). On the origin and suddenness of absences in genetic absence models. Clin. EEG Neurosci. 42, 83–97. 10.1177/155005941104200209 21675598

[B116] Veinante P. Lavallee P. Deschenes M. (2000). Corticothalamic projections from layer 5 of the vibrissal barrel cortex in the rat. J. Comp. Neurol. 424, 197–204. 10.1002/1096-9861(20000821)424:2<197::AID-CNE1>3.0.CO;2-6 10906697

[B117] Vergnes M. Marescaux C. Depaulis A. Micheletti G. Warter J. M. (1987). Spontaneous spike and wave discharges in thalamus and cortex in a rat model of genetic petit mal-like seizures. Exp. Neurol. 96, 127–136. 10.1016/0014-4886(87)90174-9 3104077

[B118] Vinck M. Van Wingerden M. Womelsdorf T. Fries P. Pennartz C. M. (2010). The pairwise phase consistency: a bias-free measure of rhythmic neuronal synchronization. Neuroimage 51, 112–122. 10.1016/j.neuroimage.2010.01.073 20114076

[B119] Westmijse I. Ossenblok P. Gunning B. van Luijtelaar G. (2009). Onset and propagation of spike and slow wave discharges in human absence epilepsy: a MEG study. Epilepsia 50, 2538–2548. 10.1111/j.1528-1167.2009.02162.x 19519798

[B120] Zheng T. W. O'Brien T. J. Morris M. J. Reid C. A. Jovanovska V. O'brien P.. (2012). Rhythmic neuronal activity in S2 somatosensory and insular cortices contribute to the initiation of absence-related spike-and-wave discharges. Epilepsia 53, 1948–1958. 10.1111/j.1528-1167.2012.03720.x 23083325

